# AlCl_3_·6H_2_O-Catalyzed Friedel-Crafts Alkylation of Indoles by the *para*-Quinone Methide Moiety of Celastrol

**DOI:** 10.3390/molecules22050742

**Published:** 2017-05-16

**Authors:** Yi Zhu, Ziwen Chen, Zhenfei Huang, Siwei Yan, Zhuoer Li, Hu Zhou, Xiaokun Zhang, Ying Su, Zhiping Zeng

**Affiliations:** 1 School of Pharmaceutical Sciences, Fujian Provincial Key Laboratory of Innovative Drug Target Research, Xiamen University, Xiamen 361102, China; yizhu@stu.xmu.edu.cn (Y.Z.); chenziwen@stu.xmu.edu.cn (Z.C.); huangzhenfei@stu.xmu.edu.cn (Z.H.); yansiwei@stu.xmu.edu.cn (S.Y.); lizhuoer@stu.xmu.edu.cn (Z.L.); huzhou@xmu.edu.cn (H.Z.); xzhang@sbpdiscovery.org (X.Z.); 2 Sanford Burnham Prebys Medical Discovery Institute, 10901 N. Torrey Pines Road, La Jolla, CA 92037, USA

**Keywords:** celastrol, indole, Friedel-Crafts alkylation, AlCl_3_·6H_2_O

## Abstract

A classical Friedel-Crafts alkylation of different indoles catalyzed by AlCl_3_·6H_2_O has been developed for a well-known important natural product, celastrol, resulting in a series of derivatives for further biological evaluation. The catalyst loading was reduced to 5 mol %, the reaction proceeds at ambient temperature and reaction time is only 3 h. The product yields range from 20% to 99%. A reaction mechanism is also proposed, based on our experiment results.

## 1. Introduction

Celastrol is a traditional Chinese medicine, first extracted from *Tripterygium wilfordii* Hook F. (also known as Thunder of God Vine or by the Chinese name ‘Lei Gong Teng”), which belongs to the Celastraceae family of plant species [1]. Structural determination revealed that celastrol is a pentacyclic triterpenoid possessing a chemically active *para*-quinone methide (pQM). This functional group could interact with DNA residues [2,3] or target proteins [4] by π-π stacking, hydrophobic interactions, or by forming hydrogen bonds and even covalent bonds [5,6]. Therefore, celastrol has been shown to be effective against many human diseases and also to act via many targets in various cells [7]. For examples, celastrol could suppress the NF-κB (nuclear factor kappa-light-chain-enhancer of activated B cells) activation by targeting cysteine 179 in the IKK (IκB kinase) and showed anti-inflammatory and anti-tumor activities in animal models [8]. Additionally, it could disrupt Hsp90 (heat shock protein 90) and Cdc37 (cell division cycle 37) interaction [9] by binding to the C-terminal domain of Hsp90; it could also induce apoptosis in multiple tumor cells by activating c-Jun N-terminal kinase and suppressing PI3K/Akt (Phosphatidylinositol-4,5-bisphosphate 3-kinase/Protein kinase B) signaling pathways [10]. Recently, it was reported that celastrol could increase leptin sensitivity and is a powerful anti-obesity agent in mouse model [11]. Moreover, celastrol was also found to be a potent Aβ lowering compound by reducing the β-cleavage of APP (Amyloid precursor protein) in a transgenic mouse model of Alzheimer’s disease [12,13]. Taken together, celastrol may be a multiple-target bioactive natural product for drug discovery.

**Scheme 1:**
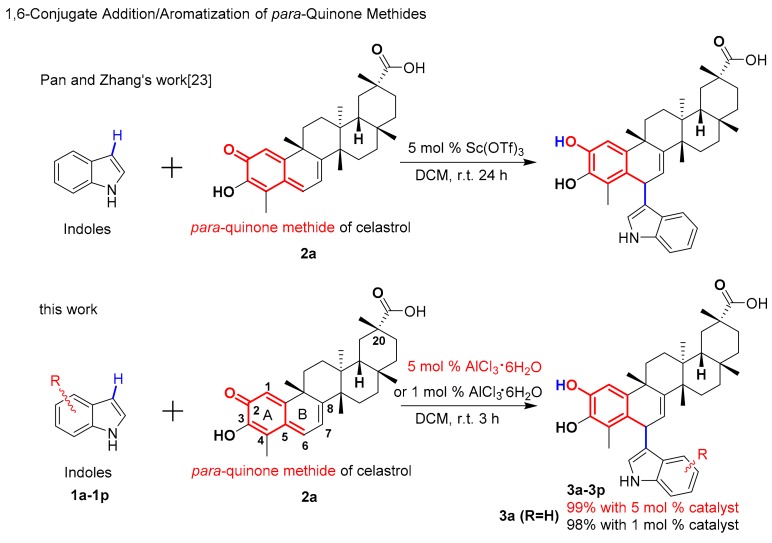
Preparation of C_6_-indole-substituted celastrol derivatives.

Though celastrol appears to be a promising drug lead, it possesses some undesirable pharmacologic properties such as high toxicity [14,15] and poor water solubility and stability [16,17]. More and more medicinal chemistry efforts are being made to design analogues or derivatives of celastrol in order to improve its drug-like properties [18,19,20,21,22,23,24]. So far, chemical modifications of celastrol have largely focused on standard esterifications or amidifications of the C_20_-carboxylic acid, esterifications or amidifications of the C_3_-hydroxyl group at ring A and C–C or C–S bond formation by C_6_ Michael addition on ring B (see Scheme 1, compound **2a**).

It is worth noting that in Pan and Zhang’s work [23], two C_6_-indole substituted celastrols could enhance antiproliferative activity against human hepatocellular carcinoma Bel7402 cancer cells from 1.55 μM (celastrol as control) to 0.01–0.02 μM. However, the formation of C–C bond between indoles and celastrol is inefficient (43–88%), and the catalyst used, scandium(III) triflate, is an extremely active Lewis acid. In addition, scandium(III) triflate is toxic [25] for large-scale production of C_6_-substituted celastrol derivatives. Therefore, the development of new C–C formation methods is imperative for the lead optimization activities of C_6_-substituted celastrol analogs. Because celastrol possesses a normal *para*-quinone methide moiety (ring A and part of ring B), C_6_-substituted celastrol could be produced by 1,6-conjugate addition of nucleophilic reagents such as indoles to this celestrol methide. Recently, several reports have revealed that the nucleophilic Michael addition could be catalyzed by 30 mol % of Fe(acac)_3_ (Tris(acetylacetonato)iron(III)) [26], copper-Fe [27], and even a pure organic chiral ligand [28], resulting in excellent yield, high stereoselectivity and enantioselectivity. However, none of these methods have been tried in the chemical modification of celastrol. In this work, we report our development of one novel, efficient and convenient method for the Michael-type Friedel-Crafts addition of indoles to the *para*-quinone methide of celastrol.

## 2. Results and Discussion

Firstly, we tested if indole could undergo Friedel-Crafts alkylation by the *para*-quinone methide of celastrol without metal salt catalysis. The result showed that the yield was only 2% when the reaction was carried out in dichloromethane (DCM) at room temperature for 12 h (Table 1, Entry 1), indicating that this reaction is inefficient and maybe is a self-catalyzed by the carboxyl acid group of celastrol. It was reported that palladium salts could catalyze Friedel-Crafts alkylation [29], so two salts, tetrakis(triphenylphosphine)palladium(0) (Table 1, Entry 2) and palladium(II) acetate (Table 1, Entry 3) were tried under the same reaction conditions and the yield was improved slightly, from 2% to 12% and 28%, respectively, therefore we can conclude that palladium salts, while somewhat better, are not suitable for catalyzing the Friedel-Crafts alkylation between indole and celastrol. We then tested a series of inorganic Lewis acids (Table 1, Entries 4–10) for the reason that they are good at accelerating Friedel-Crafts reactions [30]. We found that aluminum chloride hexahydrate could raise the yield up to 99% when the reaction was run at room temperature in DCM for 12 h (Table 1, Entry 10). Other metal chlorides such as magnesium chloride hexahydrate (Table 1, Entry 7), zinc chloride (Table 1, Entry 8), and iron(III) chloride hexahydrate (Table 1, Entry 9) could also efficiently catalyze the Friedel-Crafts alkylation between indole and celastrol (yields: 64%, 80% and 93%, respectively). On the other hand, aluminium hydroxide failed to catalyze this reaction (Table 1, Entry 5) and the catalytic efficiency of both iron(III) phosphate hexahydrate (Table 1, Entry 4) and hydrated aluminium sulphate (Table 1, Entry 6) was marginally low. Hence, aluminum chloride hexahydrate was selected as the ideal catalyst for our system.

In order to investigate the solvent effect on this reaction, a protic solvent (MeOH, Table 1, Entry 11) was used to replace DCM, but the yield of **3a** dropped to 95%. We also tried to increase the solvent polarity (Table 1, Entries 12–15). However, none of the yields retained in high level, dropping to 84–93%. Therefore, DCM was the best solvent for this catalytic process. We also investigated the effect of the reaction time by reducing it from 12 h to 6 h, 3 h, 1.5 h and 0.5 h (Table 1, Entries 10, 16–19), and found that our reaction was finished after 3 h.

Futhermore, the amount of reactant indole could be reduced from 2 eq to 1.2 eq (Table 1, Entries 17, 20–21) without apparent loss of yield (99%). Finally, the amount of catalyst, aluminum chloride hexahydrate, could be reduced to 1 mol % (Table 1, Entries 21, 23–24) and the yield of the product stayed high. However, for the reason that aluminum chloride hexahydrate is cheap and for the convenient weighing of this catalyst, the loading amount was chosen as 5 mol % (0.53 mg). Taken together, the optimal reaction conditions would be that celastrol (1 eq), indole (1.2 eq), catalyst aluminum chloride hexahydrate (5 mol %), solvent (DCM, 1 mL) reaction time (3 h) and room temperature. For the convenient calculation of isolated yields, the amount of celastrol was increased from 0.045 mmol to 0.222 mmol (see the Experimental Section).

**Scheme 2:**
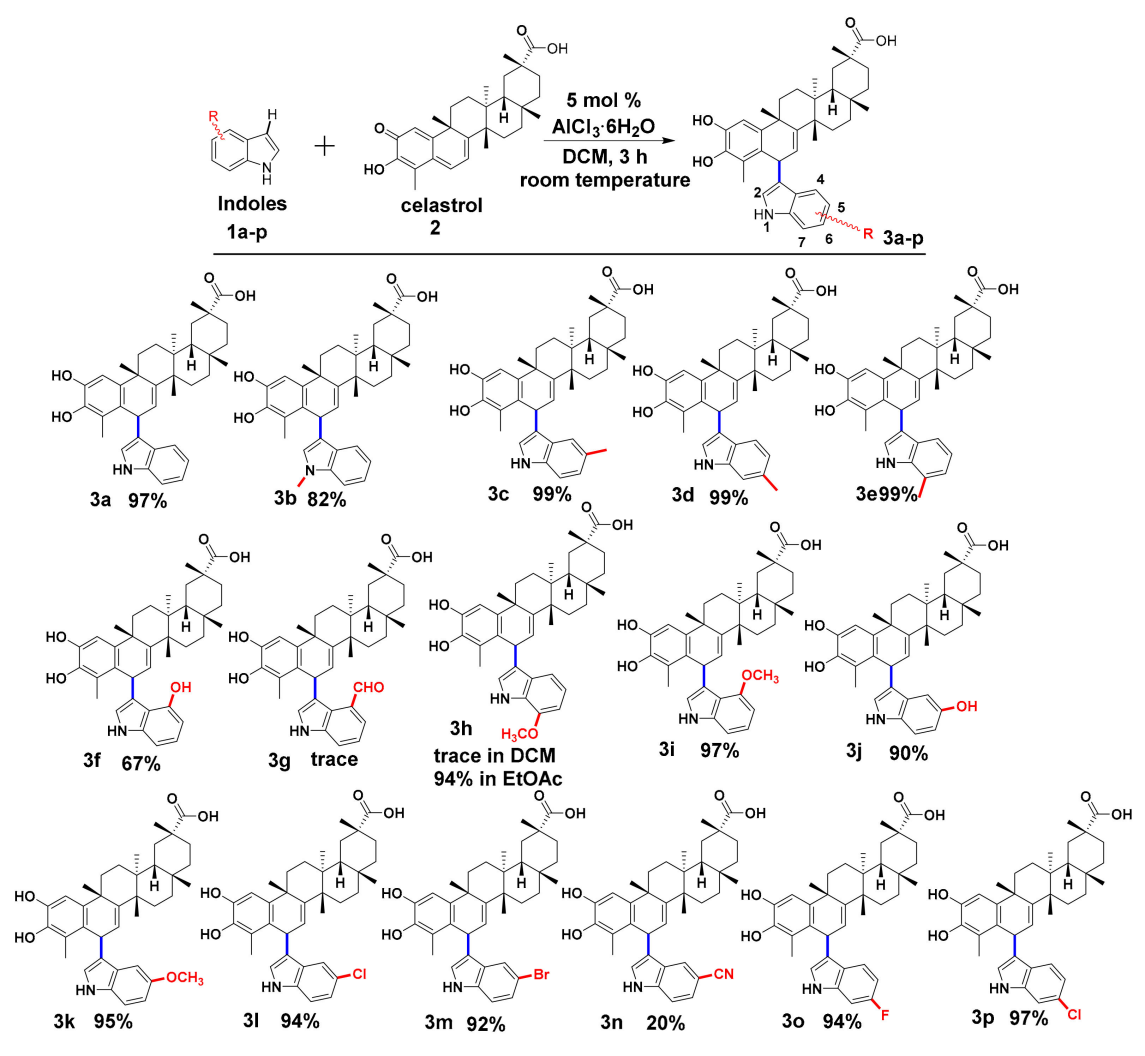
Reaction scope and limitations using indoles as the substrates.

With an optimized method in hand, we examined the scope and versatility of the reaction of different indole derivatives with celastrol (see Scheme 2). Firstly, a methyl group was introduced into different positions of the indole ring (compounds **1b**–**1e**). It seems that increasing steric hindrance at positions 5, 6 and 7 of the benzene ring moiety of the indole (compounds **1c**–**1e**) had no negative effect on the yield (99% for **3c** to **3e**). However, methylation of the nitrogen atom of the indole ring dropped the yield of **3b** to 82%. When the indole ring was hydroxylated at position 4 (**1f**) or 5 (**1j**), the yield of its alkylation by the *para*-quinone methide moiety of celastrol was reduced to 67% (**3f**) and 90% (**3j**), respectively. It is interesting to note that methylation of this hydroxyl group (compounds **1i** and **1k**) restored the yield to 95% (**3k**) and 97% (**3i**). The effect of a halogen substituent group such as fluoride, choloride and bromide at different positions of the benzene ring moiety of the indole (compounds **1l**–**1n**, **1o**–**1p**) was quite similar to that of a methoxyl group (yield 92–97%, see **3l**–**3n**, **3o**–**3p**).

On the other hand, the incorporation of an electron-withdrawing group such as a cyano group at the C_5_ position of the indole ring sharply decreased the yield (**3n**, 20%). Moreover, an aldehyde group, a stronger electron-withdrawing group than a cyano group, only produced traces of product **3g**, implying that electron-withdrawing groups on the indole ring have a negative effect on the Friedel-Crafts alkylation of indoles by the *para*-quinone methide moiety of celastrol.

**Scheme 3:**
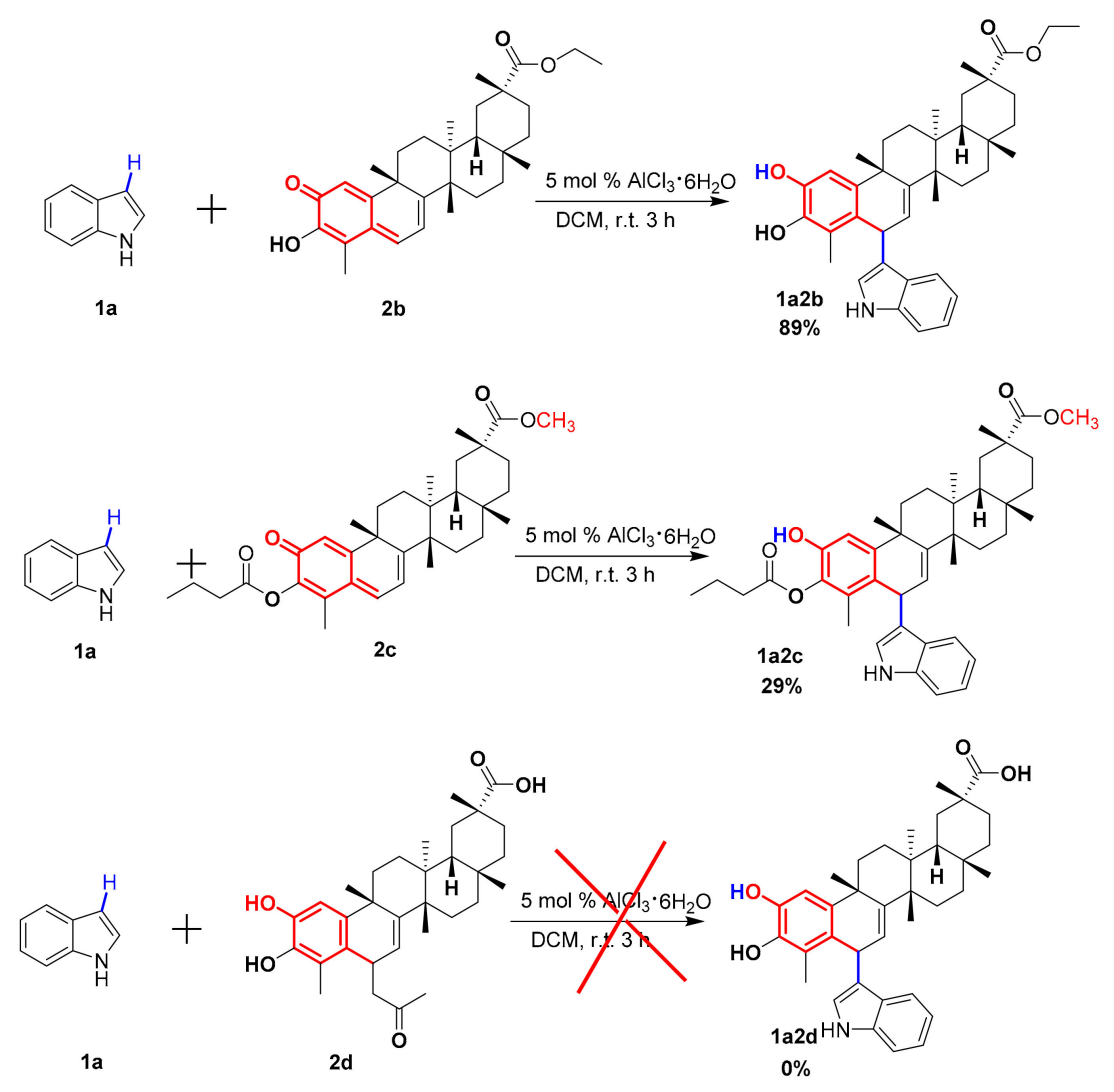
Reaction scope using different celastrol derivatives as the substrates.

Furthermore, in order to evaluate the influences of the carboxyl and hydroxyl groups of celastrol in this reaction, we synthesized compounds **2a**–**2c** (Scheme 3). The results showed that ethylation of the carboxylic acid of celastrol reduced the catalytic efficiency of aluminum chloride hexahydrate, leading to a yield of only 89% (Scheme 3, **1a2b**). Furthermore, if the hydroxyl group was blocked, the reaction yield further dropped to 29% (Scheme 3, **1a2c**). Additionally, Friedel-Crafts addition did not occur when the C6 position of celastrol was blocked by a sterically hindered group such as a propan-2-one group (Scheme 3, **1a2d**).

According to the reaction mechanism, a 1,2-, 1,4-, and 1,6-addition of dienone could happen for celastrol. However, our experimental results show that the 1,6-conjugate addition of *para*-quinone methide moiety of celastrol by C_3_ position of indole occurs. The reason is that the hindrance of the C_4_-position in ring A of celastrol is larger than at the C_2_ and C_6_ position which make 1,4-addition more difficult (see 3D structure of celastrol in Figure S1). Celastrol could produce five carbocationic intermediates (see Scheme S1) catalyzed by AlCl_3_·6H_2_O. The yield of reaction products depends on the stability of these five carbocationic intermediates. Apparently, INS4 (intermediate 4, see Scheme S1) is the most stable one and the easiest to attack an indole ring, so the 1,6-addition product of celastrol dominates this reaction. The reaction progress was monitored by TLC (thin-layer chromatography) and HPLC (high-performance liquid chromatography) and only one product was observed from start to end, so other regioisomers did not appear.

Secondly, the regioselectivity of the addition to the indole moiety depends on the stability of indole intermediates generated by the attack of C_6_ carboncationic celastrol (see Scheme S2). Seven intermediates can be produced in this step. According to our quantum mechanics calculation, the C_3_ position of indole possesses a more negative charge (−0.383) than other carbon atoms (C_2_, C_4_, C_5_, C_6_, C_7_) and hence is easiest to be attacked by C_6_-celastrol. In the other hand, the carboncationic indole intermediate INS2 (intermediate 2, see Scheme S2) has two forms (indole-INS2-1 and indole-INS2-2) that stabilize the positive charge, which makes the alkylation of C_3_ prone to occur. That is the origin of the indole regioselectivity.

**Scheme 4:**
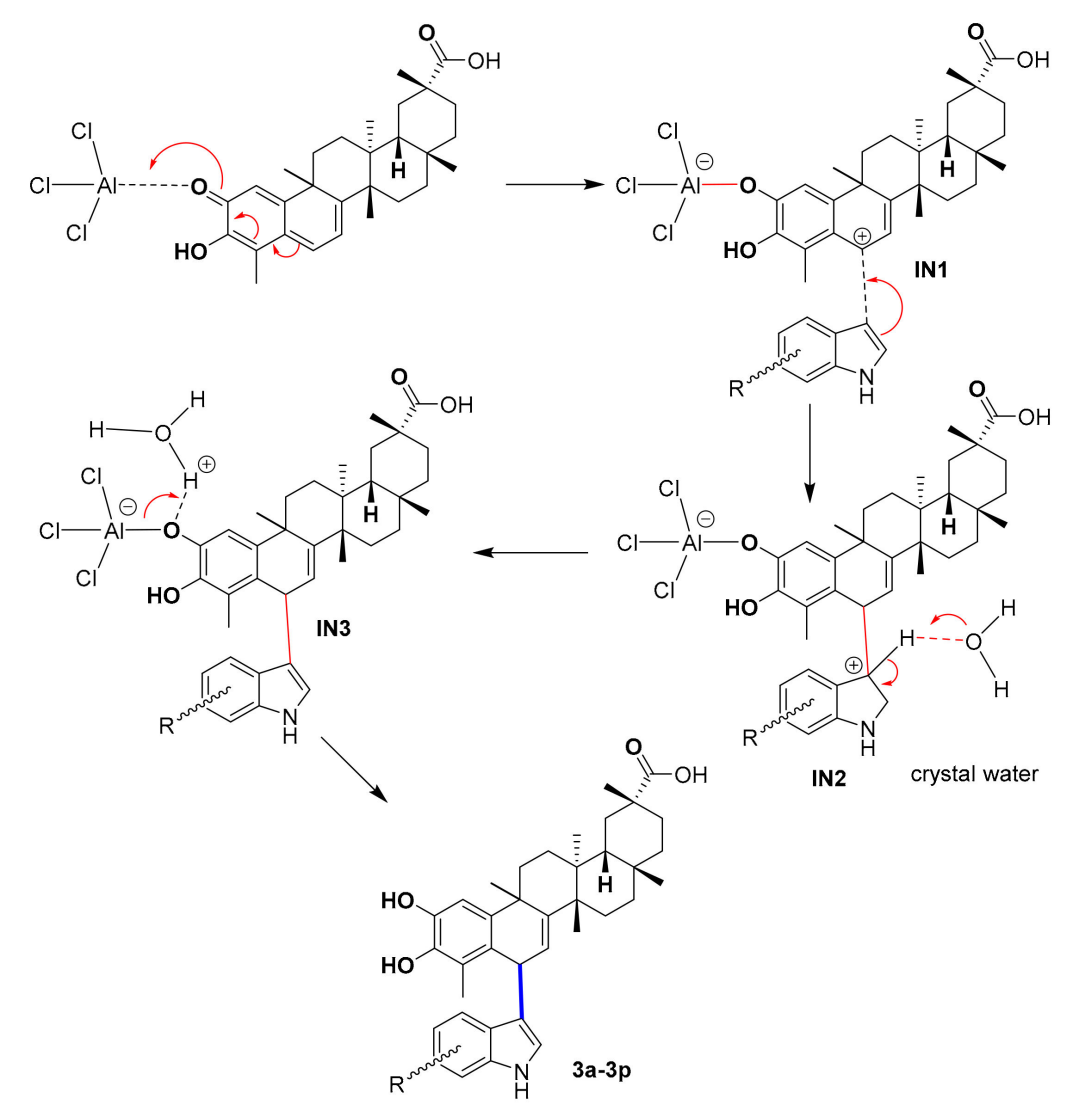
The proposed mechanism for Friedel-Crafts Alkylation.

According to the results, a possible mechanism of this special Friedel-Crafts reaction is proposed in Scheme 4. As aluminium trichloride is a strong Lewis acid, the oxygen atom from the carbonyl group in ring A of celastrol could first form a coordinate bond with the electron-deficient aluminium [31], resulting in an electron migration from C_6_ to aluminium and then the formation of a cationic species (intermediate IN1). Subsequently, electrophilic addition to the indoles by IN1 to form an arenium ion (also named the Wheland intermediate [32]) (IN2) would occur. Because of the partial elimination of aromaticity, this intermediate is very unstable. Therefore, one of its protons could be removed by a crystallization water of the aluminium trichloride catalyst, forming intermediate IN3. Finally, the chelated aluminium trichloride would be easily removed from IN3 assisted by a protonated crystallization water produced from IN2, yielding the final product.

## 3. Materials and Methods

### 3.1. General Information

All the reagents were purchased from Asia Alfa Aesar (Shanghai, China) and J&K Chemical (Beijing, China) and used without further purification. ^1^H-NMR (600 MHz) and ^13^C-NMR (151 MHz) spectra were measured on a 600M spectrometer (Bruker in Asia Pacific, Beijing, China) with CDCl_3_ or DMSO-*d*_6_ (dimethyl sulfoxide-*d*_6_) as solvents and tetramethylsilane (TMS) as an internal standard. Chemical shifts were reported in units (ppm) by assigning TMS resonance in the ^1^H spectrum as 0.00 ppm and CDCl_3_ resonance in the ^13^C spectrum as 77.0 ppm. All coupling constants (*J* values) were reported in Hertz (Hz). Chemical shifts of common trace ^1^H-NMR impurities (ppm): H_2_O: 1.56, CHCl_3_: 7.26. All new compounds were further characterized by HRMS (high resolution mass spectroscopy), using a Q-Exactive apparatus (ThermoFisher, Shanghai, China). Column chromatography was performed on 300–400 mesh silica gel. The CAS numbers of the known compounds are listed under the corresponding entry. The spectroscopic data of the known compounds matched the data reported in the corresponding references.

### 3.2. Screening Method for the Optimization of Friedel-Crafts Alkylation of Indoles with Celestrol

#### 3.2.1. HPLC Parameters

Mobile phase: acetonitrile: H_2_O (0.2% H_3_PO_4_) = 75:25. Column: 4.6 mm × 250 mm BDS Hypersil C18 Column (5 µm particle size) (Thermo Scientific, Shanghai, China). Instrument: LC-20AT (Shimadzu Corporation, Kyoto, Japan). Detector: SPD-20A (Shimadzu Corporation). The retention time of **3a** is 10.7 min.

#### 3.2.2. Establishment of the Standard Curve

Preparation for the standard solution: 50 mg of product **3a** was dissolved with acetonitrile in a 25 mL volumetric flask, obtaining a solution of concentration for 2.0 mg/mL. This method was repeated to get the other solutions of different concentrations, 1.0 mg/mL, 0.5 mg/mL, 0.25 mg/mL, 0.125 mg/mL. The results are shown in Table S1 and Figures S2–S7.

#### 3.2.3. General Procedure for the Screening Method

Celastrol (0.045 mmol, 20 mg), indole (0.045–0.090 mmol) was dissolved by 1 mL solvent in a 10 mL closed tube. After intensive stirring at room temperature, the solution was added with metal catalyst (1–10 mmol %) and the reaction was kept for 3 h. Without any postprocessing, the mixture was diluted with acetonitrile in a 25 mL volumetric flask. The yield of product was immediately determined by HPLC (see Scheme 5). HPLC spectra can be found in Figures S8–S31.

### 3.3. General Procedure for the Friedel-Crafts Alkylation

Celastrol (**2a**, 0.222 mmol, 100 mg) and indole (**3a**, 0.266 mmol, 31 mg) were dissolved in dichloromethane (DCM, 2 mL) in a 10 mL closed tube. After intensive stirring at room temperature, the metal catalyst AlCl_3_·6H_2_O (5 mol %, 2.7 mg) was added to the solution and the reaction mixture was stirred for 3 h. When the reaction was finished, pure water (20 mL) was added to stop the reaction and subsequently the aqueous phase was separated and extracted with ethyl acetate (20 mL) three times. The organic layers were combined and dried with anhydrous sodium sulfate. The solvent was removed in vacuo, giving a crude mixture which was purified by flash chromatography on silica column (hexane/ethyl acetate/AcOH = 4:1:0.005) to give the pure products **3a**–**3q**, **1a2b**, **1a2c**. NMR spectra for all compounds can be found in Supplementary Materials.

### 3.4. Physical, Analytical, and Spectral Data of Synthesized Compounds

*(2R,4aS,6aS,12bR,14aS,14bR)-10,11-Dihydroxy-8-(1H-indol-3-yl)-2,4a,6a,9,12b,14a-hexamethyl-1,2,3,4,4a,5,6,6a,8,12b,13,14,14a,14b-tetradecahydropicene-2-carboxylic acid* (**3a**): CAS: 2022987-39-7. Starting from 100 mg of celastrol (**2a**), compound **3a** (122.2 mg, 97%) was obtained as a wine-red solid according to the abovementioned procedure. ^1^H-NMR (CDCl_3_) δ 0.73 (br. s., 3H), 0.87–0.90 (m, 1H), 0.95–1.00 (m, 3H), 1.01 (br. s., 3H), 1.14 (br. s., 3H), 1.19 (t, *J* = 7.06 Hz, 1H), 1.25–1.27 (m, 1H), 1.34 (br. s., 3H), 1.42–1.57 (m, 4H), 1.57–1.76 (m, 4H), 1.90 (s, 3H), 1.99–2.07 (m, 2H), 2.10–2.17 (m, 1H), 2.40 (d, *J* = 15.04 Hz, 1H), 4.90 (d, *J* = 5.69 Hz, 1H), 6.21 (d, *J* = 6.24 Hz, 1H), 6.23 (br. s., 1H), 6.79 (br. s., 1H), 7.11 (t, *J* = 7.43 Hz, 1H), 7.16 (t, *J* = 7.43 Hz, 1H), 7.28 (d, *J* = 7.89 Hz, 1H), 7.75 (d, *J* = 7.70 Hz, 1H), 7.88 (br. s., 1H). ^13^C-NMR (CDCl_3_) δ 11.53, 18.84, 21.93, 28.89, 29.63, 29.75, 30.40, 30.54, 30.72, 31.55, 32.84, 34.62, 35.50, 36.74, 36.94, 37.76, 40.35, 43.62, 44.28, 108.93, 111.30, 119.09, 119.28, 120.23, 121.55, 121.58, 121.67, 127.10, 127.84, 136.49, 139.92, 142.17, 142.83, 147.48, 184.30. HRMS (ESI) calcd for C_37_H_44_NO_4_^−^ [M^−^]: 566.2376; found: 566.2376.

*(2R,4aS,6aS,12bR,14aS,14bR)-10,11-Dihydroxy-2,4a,6a,9,12b,14a-hexamethyl-8-(1-methyl-1H-indol-3-yl)-1,2,3,4,4a,5,6,6a,8,12b,13,14,14a,14b-tetradecahydropicene-2-carboxylic acid* (**3b**): Starting from 100 mg of celastrol (**2a**), compound **3b** (106.1 mg, 82%) was obtained as a purple solid according to the above-mentioned procedure. ^1^H-NMR (DMSO-*d*_6_) δ 0.72 (s, 3H), 0.82–0.87 (m, 1H), 0.95 (s, 3H), 1.00 (s, 3H), 1.10 (s, 3H), 1.20–1.36 (m, 3H), 1.36 (s, 3H), 1.44 (d, *J* = 8.07 Hz, 1H), 1.50–1.76 (m, 6 H), 1.80 (s, 3H), 1.98–2.09 (m, 3H), 2.34 (d, *J* = 15.41 Hz, 1H), 3.58 (s, 3H), 4.84 (d, *J* = 6.05 Hz, 1H), 6.11 (d, *J* = 6.42 Hz, 1H), 6.31 (s, 1H), 6.75 (s, 1H), 6.98 (t, *J* = 7.52 Hz, 1H), 7.10 (t, *J* = 7.70 Hz, 1H), 7.30 (d, *J* = 8.25 Hz, 1H), 7.62 (d, *J* = 7.89 Hz, 1H), 7.86 (br. s., 1H), 8.97 (br. s., 1H), 12.11 (br. s., 1H). ^13^C-NMR (DMSO-*d*_6_) δ 11.97, 18.48, 22.30, 29.15, 29.93, 30.41, 30.53, 30.60, 31.46, 31.83, 32.61, 32.91, 34.95, 35.23, 35.56, 35.88, 36.79, 36.88, 37.80, 40.45, 43.55, 44.33, 108.85, 110.04, 118.63, 118.75, 119.43, 121.21, 121.33, 122.56, 126.05, 126.31, 127.38, 137.09, 140.89, 141.46, 144.08, 146.97, 179.99. HRMS (ESI) calcd for C_38_H_46_NO_4_^−^ [M^−^]: 580.3432; found: 580.3418.

*(2R,4aS,6aS,12bR,14aS,14bR)-10,11-Dihydroxy-2,4a,6a,9,12b,14a-hexamethyl-8-(5-methyl-1H-indol-3-yl)-1,2,3,4,4a,5,6,6a,8,12b,13,14,14a,14b-tetradecahydropicene-2-carboxylic acid* (**3c**): CAS: 2022987-43-3. Starting from 100 mg of celastrol (**2a**), compound **3c** (127.5 mg, 99%) was obtained as a wine-red solid according to the abovementioned procedure. ^1^H-NMR (DMSO-*d*_6_) δ 0.72 (s, 3H), 0.84–0.88 (m, 1H), 0.98 (s, 3H), 1.02 (s, 3H), 1.11 (s, 3H), 1.26–1.32 (m, 2H), 1.37 (s, 3H), 1.45 (d, *J* = 8.1 Hz, 1H), 1.51–1.62 (m, 4H), 1.63–1.75 (m, 3H), 1.78 (s, 3H), 2.00–2.08 (m, 3H), 2.34 (d, *J* = 15.6 Hz, 1H), 2.39 (s, 3H), 4.79 (d, *J* = 6.1 Hz, 1H), 6.10 (d, *J* = 6.2 Hz, 1H), 6.23 (s, 1H), 6.73 (s, 1H), 6.88 (d, *J* = 8.3 Hz, 1H), 7.19 (d, *J* = 8.3 Hz, 1H), 7.45 (s, 1H), 7.85 (br. s., 1H), 8.94 (br. s., 1H), 10.45 (s, 1H), 12.08 (br. s., 1H). ^13^C-NMR (DMSO-*d*_6_) δ 11.89, 18.46, 21.88, 22.28, 29.15, 29.92, 30.39, 30.50, 30.61, 31.44, 31.83, 32.92, 34.93, 35.25, 35.56, 35.78, 36.74, 36.90, 37.80, 39.91, 43.54, 44.32, 108.75, 111.65, 118.66, 118.81, 121.13, 121.96, 122.53, 122.84, 126.38, 127.01, 127.33, 135.05, 140.78, 141.43, 144.00, 146.73, 180.04. HRMS (ESI) calcd. for C_38_H_46_NO_4_^−^ [M^−^]: 580.3432; found: 580.3417.

*(2R,4aS,6aS,12bR,14aS,14bR)-10,11-Dihydroxy-2,4a,6a,9,12b,14a-hexamethyl-8-(6-methyl-1H-indol-3-yl)-1,2,3,4,4a,5,6,6a,8,12b,13,14,14a,14b-tetradecahydropicene-2-carboxylic acid* (**3d**): CAS: 2022987-46-6. Starting from 100 mg of celastrol (**2a**), compound **3d** (128 mg, 99%) was obtained as a wine-red solid according to the abovementioned procedure. ^1^H-NMR (DMSO-*d*_6_) δ 0.71 (s, 3H), 0.83–0.87 (m, 1H), 0.95 (s, 3H), 1.00 (s, 3H), 1.10 (s, 3H), 1.21–1.29 (m, 2H), 1.32 (s, 3H), 1.34–1.39 (m, 2H), 1.44 (d, *J* = 8.1 Hz, 1H), 1.49–1.61 (m, 3H), 1.62–1.74 (m, 2H), 1.79 (s, 3H), 1.96–2.07 (m, 3H), 2.33 (d, *J* = 15.4 Hz, 1H), 2.36 (s, 3H), 4.78 (d, *J* = 6.1 Hz, 1H), 6.12 (d, *J* = 6.4 Hz, 1H), 6.21 (s, 1H), 6.72 (s, 1H), 6.78 (d, *J* = 8.1 Hz, 1H), 7.08 (s, 1H), 7.46 (d, *J* = 8.1 Hz, 1H), 7.84 (s, 1H), 8.94 (br. s., 1H), 10.41 (s, 1H), 12.06 (br. s., 1H). ^13^C-NMR (DMSO-*d*_6_) δ 11.87, 18.48, 21.83, 22.31, 29.13, 29.92, 30.38, 30.50, 30.60, 31.82, 32.89, 34.93, 35.34, 35.51, 35.69, 36.76, 36.89, 37.77, 43.54, 44.31, 108.77, 111.73, 118.98, 119.03, 120.43, 121.19, 122.70, 125.03, 126.33, 130.15, 137.15, 140.83, 141.37, 143.96, 146.81, 180.00. HRMS (ESI) calcd. for C_38_H_46_NO_4_^−^ [M^−^]: 580.3432; found: 580.3419.

*(2R,4aS,6aS,12bR,14aS,14bR)-10,11-Dihydroxy-2,4a,6a,9,12b,14a-hexamethyl-8-(7-methyl-1H-indol-3-yl)-1,2,3,4,4a,5,6,6a,8,12b,13,14,14a,14b-tetradecahydropicene-2-carboxylic acid* (**3e**): CAS: 2022987-40-0. Starting from 100 mg of celastrol (**2a**), compound **3e** (128 mg, 99%) was obtained as a purple solid according to the abovementioned procedure. ^1^H-NMR (DMSO-*d*_6_) δ 0.71 (s, 3H), 0.83–0.86 (m, 1H), 0.95 (s, 3H), 1.00 (s, 3H), 1.10 (s, 3H), 1.22–1.29 (m, 2H), 1.33 (s, 3H), 1.34–1.39 (m, 2H), 1.44 (d, *J* = 7.9 Hz, 1H), 1.49–1.60 (m, 3H), 1.62–1.74 (m, 2H), 1.79 (s, 3H), 1.96–2.07 (m, 3H), 2.33 (d, *J* = 15.4 Hz, 1H), 2.40 (s, 3H), 4.81 (d, *J* = 6.1 Hz, 1H), 6.13 (d, *J* = 6.6 Hz, 1H), 6.26 (s, 1H), 6.73 (s, 1H), 6.84 (d, *J* = 7.2 Hz, 1H), 6.88 (t, *J* = 7.4 Hz, 1H), 7.45 (d, *J* = 7.9 Hz, 1H), 7.86 (br. s., 1H), 8.96 (br. s., 1H), 10.56 (s, 1H), 12.08 (br. s., 1H). ^13^C-NMR (DMSO-*d*_6_) δ 11.89, 17.20, 18.47, 22.32, 29.13, 29.91, 30.38, 30.49, 30.60, 31.44, 31.82, 32.89, 34.92, 35.39, 35.52, 35.69, 36.76, 36.87, 37.77, 43.54, 44.30, 108.78, 116.91, 118.92, 119.70, 120.91, 121.18, 121.61, 121.77, 122.62, 126.26, 126.72, 136.20, 140.87, 141.40, 143.98, 146.89, 180.00. HRMS (ESI) calcd. for C_38_H_46_NO_4_^−^ [M^−^]: 580.3432; found: 580.3420.

*(2R,4aS,6aS,12bR,14aS,14bR)-10,11-Dihydroxy-8-(4-hydroxy-1H-indol-3-yl)-2,4a,6a,9,12b,14a-hexa-methyl-1,2,3,4,4a,5,6,6a,8,12b,13,14,14a,14b-tetradecahydropicene-2-carboxylic acid* (**3f**): Starting from 100 mg of celastrol (**2a**), compound **3f** (86.8 mg, 67%) was obtained as a black brown solid according to the abovementioned procedure. ^1^H-NMR (DMSO-*d*_6_) δ 0.70 (s, 3H), 0.82–0.85 (m, 1H), 0.98 (s, 3H), 1.01 (s, 3H), 1.10 (s, 3H), 1.22–1.37 (m, 4H), 1.38 (s, 3H), 1.45 (d, *J* = 7.7 Hz, 1H), 1.48–1.61 (m, 3H), 1.64 (d, *J* = 12.8 Hz, 1H), 1.68–1.78 (m, 2H), 1.81 (s, 3H), 2.01–2.08 (m, 2H), 2.33 (d, *J* = 15.4 Hz, 1H), 5.05 (d, *J* = 5.9 Hz, 1H), 5.95 (s, 1H), 6.31 (d, *J* = 6.1 Hz, 1H), 6.34 (d, *J* = 7.5 Hz, 1H), 6.70 (s, 1H), 6.73 (d, *J* = 7.9 Hz, 1H), 6.80 (t, *J* = 7.7 Hz, 1H), 7.79 (s, 1H), 8.88 (s, 1H), 9.41 (s, 1H), 10.36 (s, 1H), 12.09 (br. s., 1H). ^13^C-NMR (DMSO-*d*_6_) δ 11.69, 14.11, 17.96, 21.86, 28.79, 29.49, 30.04, 30.06, 30.18, 31.41, 32.47, 34.53, 35.17, 35.30, 35.53, 36.26, 36.47, 37.37, 43.02, 43.91, 102.81, 103.05, 108.22, 116.34, 119.35, 119.42, 120.63, 121.59, 124.01, 126.61, 138.53, 140.54, 140.96, 143.46, 145.06, 151.80, 179.57. HRMS (ESI) calcd. for C_37_H_44_NO_5_^−^ [M^−^]: 582.3225; found: 582.3219.

*(2R,4aS,6aS,12bR,14aS,14bR)-10,11-Dihydroxy-8-(7-methoxy-1H-indol-3-yl)-2,4a,6a,9,12b,14a-hexa-methyl-1,2,3,4,4a,5,6,6a,8,12b,13,14,14a,14b-tetradecahydropicene-2-carboxylic acid* (**3h**): Starting from 100 mg of celastrol (**2a**), compound **3h** (124.9 mg, 94%) was obtained as a purplish grey solid according to the abovementioned procedure using ethyl acetate as solvent. ^1^H-NMR (DMSO-*d*_6_) δ 0.71 (s, 3H), 0.86 (d, *J* = 9.9 Hz, 1H), 0.96 (s, 3H), 1.01 (s, 3H), 1.10 (s, 3H), 1.22–1.29 (m, 2H), 1.32 (s, 3H), 1.34–1.40 (m, 2H), 1.45 (d, *J* = 8.1 Hz, 1H), 1.50–1.61 (m, 3H), 1.62–1.75 (m, 2H), 1.79 (s, 3H), 1.96–2.08 (m, 3H), 2.34 (d, *J* = 15.4 Hz, 1H), 3.88 (s, 3H), 4.79 (d, *J* = 5.9 Hz, 1H), 6.12 (d, *J* = 6.2 Hz, 1H), 6.18 (s, 1H), 6.62 (d, *J* = 7.7 Hz, 1H), 6.73 (s, 1H), 6.90 (t, *J* = 7.9 Hz, 1H), 7.22 (d, *J* = 7.9 Hz, 1H), 7.88 (br. s., 1H), 8.99 (br. s., 1H), 10.69 (s, 1H), 12.04 (br. s., 1H). ^13^C-NMR (DMSO-*d*_6_) δ 11.9, 18.5, 22.3, 29.1, 29.9, 30.4, 30.5, 30.6, 31.8, 32.9, 34.9, 35.4, 35.5, 35.6, 36.8, 36.9, 37.8, 43.5, 44.3, 55.4, 101.8, 108.8, 112.2, 119.2, 119.8, 121.1, 121.6, 122.6, 126.3, 126.7, 128.5, 140.8, 141.4, 144.0, 146.6, 147.0, 180.0. HRMS (ESI) calcd. for C_38_H_46_NO_5_^−^ [M^−^]: 596.3381; found: 596.3372.

*(2R,4aS,6aS,12bR,14aS,14bR)-10,11-Dihydroxy-8-(4-methoxy-1H-indol-3-yl)-2,4a,6a,9,12b,14a-hexa-methyl-1,2,3,4,4a,5,6,6a,8,12b,13,14,14a,14b-tetradecahydropicene-2-carboxylic acid* (**3i**): Starting from 100 mg of celastrol (**2a**), compound **3i** (128.4 mg, 97%) was obtained as a purplish grey solid according to the abovementioned procedure. ^1^H-NMR (DMSO-*d*_6_) δ 0.71 (s, 3H), 0.82–0.89 (m, 1H), 0.97 (s, 3H), 1.01 (s, 3H), 1.11 (s, 3H), 1.22–1.32 (m, 3H), 1.38 (s, 3H), 1.42–1.48 (m, 1H), 1.50–1.61 (m, 3H), 1.61–1.76 (m, 3H), 1.79 (s, 3H), 1.92–2.12 (m, 3H), 2.33 (d, *J* = 14.9 Hz, 1H), 3.94 (s, 3H), 5.01 (d, *J* = 14.9 Hz, 1H), 6.00 (s, 1H), 6.15–6.29 (m, 1H), 6.49 (d, *J* = 7.3 Hz, 1H), 6.71 (s, 1H), 6.84–7.07 (m, 2H), 7.82 (s, 1H), 8.93 (s, 1H), 10.54 (s, 1H), 12.11 (br. s., 1H). ^13^C-NMR (DMSO-*d*_6_) δ 11.68, 18.04, 21.89, 28.49, 29.50, 30.02, 30.08, 30.21, 31.41, 32.48, 34.49, 35.18, 35.30, 35.70, 36.29, 36.56, 37.40, 40.46, 43.02, 43.92, 55.29, 98.98, 105.04, 108.26, 116.65, 119.27, 120.04, 120.58, 121.60, 123.56, 126.52, 137.88, 140.56, 141.00, 143.51, 145.54, 154.25, 179.61. HRMS (ESI) calcd. for C_38_H_46_NO_5_^−^ [M^−^]: 596.3381; found: 596.3372.

*(2R,4aS,6aS,12bR,14aS,14bR)-10,11-Dihydroxy-8-(5-hydroxy-1H-indol-3-yl)-2,4a,6a,9,12b,14a-hexa-methyl-1,2,3,4,4a,5,6,6a,8,12b,13,14,14a,14b-tetradecahydropicene-2-carboxylic acid* (**3j**): Starting from 100 mg of celastrol (**2a**), compound **3j** (116.6 mg, 90%) was obtained as a purple solid according to the abovementioned procedure. ^1^H-NMR (DMSO-*d*_6_) δ 0.75 (s, 3H), 0.84–0.90 (m, 1H), 1.01 (s, 3H), 1.03 (s, 3H), 1.13 (s, 3H), 1.20–1.34 (m, 2H), 1.38 (s, 3H), 1.41 (m, 2H), 1.48 (d, *J* = 7.7 Hz, 1H), 1.53–1.64 (m, 3H), 1.66–1.79 (m, 2H), 1.84 (s, 3H), 2.00–2.13 (m, 3H), 2.37 (d, *J* = 15.2 Hz, 1H), 4.74 (d, *J* = 5.9 Hz, 1H), 6.13 (d, *J* = 6.4 Hz, 1H), 6.22 (s, 1H), 6.63 (dd, *J* = 8.5, 2.1 Hz, 1H), 7.00 (s, 1H), 7.12 (d, *J* = 8.6 Hz, 1H), 7.84 (s, 1H), 8.62 (s, 1H), 8.94 (br. s., 1H), 10.27 (s, 1H), 12.10 (br. s., 1H). ^13^C-NMR (DMSO-*d*_6_) δ 11.90, 18.51, 22.35, 29.18, 29.95, 30.43, 30.56, 30.62, 31.84, 32.92, 34.97, 35.42, 35.55, 35.77, 36.76, 36.93, 37.80, 39.91, 43.57, 44.35, 103.23, 108.78, 111.57, 112.23, 118.31, 121.18, 122.39, 122.42, 126.41, 127.77, 131.36, 140.87, 141.40, 143.94, 146.85, 150.54, 180.03. HRMS (ESI) calcd. for C_37_H_44_NO_5_^−^ [M^−^]: 582.3225; found: 582.3212.

*(2R,4aS,6aS,12bR,14aS,14bR)-10,11-Dihydroxy-8-(5-methoxy-1H-indol-3-yl)-2,4a,6a,9,12b,14a-hexa-methyl-1,2,3,4,4a,5,6,6a,8,12b,13,14,14a,14b-tetradecahydropicene-2-carboxylic acid* (**3k**): CAS: 2022987-44-4. Starting from 100 mg of celastrol (**2a**), compound **3k** (126 mg, 95%) was obtained as a wine-red solid according to the abovementioned procedure. ^1^H-NMR (DMSO-*d*_6_) δ 0.72 (s, 3H), 0.84–0.89 (m, 1H), 0.98 (s, 3H), 1.02 (s, 3H), 1.11 (s, 3H), 1.24–1.32 (m, 2H), 1.34 (s, 3H), 1.36–1.42 (m, 2H), 1.46 (d, *J* = 8.1 Hz, 1H), 1.51–1.63 (m, 3H), 1.66 (d, *J* = 11.9 Hz, 1H), 1.69–1.78 (m, 1H), 1.80 (s, 3H), 1.97–2.08 (m, 3H), 2.34 (d, *J* = 15.4 Hz, 1H), 6.13 (d, *J* = 6.4 Hz, 1H), 6.35 (d, *J* = 2.2 Hz, 1H), 6.69 (dd, *J* = 8.6, 2.4 Hz, 1H), 6.74 (s, 1H), 6.99 (d, *J* = 2.2 Hz, 1H), 7.19 (d, *J* = 8.6 Hz, 1H), 7.85 (br. s., 1H), 8.94 (br. s., 1H), 10.45 (s, 1H), 12.10 (br. s., 1H). ^13^C-NMR (DMSO-*d*_6_) δ 11.47, 18.03, 21.87, 28.68, 29.48, 29.97, 30.06, 30.18, 31.39, 32.47, 34.49, 34.82, 35.13, 35.34, 36.32, 36.49, 37.37, 39.47, 43.13, 43.89, 55.32, 100.92, 108.35, 110.62, 112.01, 118.35, 120.80, 122.29, 122.40, 125.90, 126.81, 131.42, 140.39, 140.95, 143.59, 146.24, 152.77, 179.57. HRMS (ESI) calcd. for C_38_H_46_NO_5_^−^ [M^−^]: 596.3381; found: 596.3370.

*(2R,4aS,6aS,12bR,14aS,14bR)-8-(5-Chloro-1H-indol-3-yl)-10,11-Dihydroxy-2,4a,6a,9,12b,14a-hexamethyl-1,2,3,4,4a,5,6,6a,8,12b,13,14,14a,14b-tetradecahydropicene-2-carboxylic acid* (**3l**): Starting from 100 mg of celastrol (**2a**), compound **3l** (125 mg, 94%) was obtained as a wine-red solid according to the above-mentioned procedure. ^1^H-NMR (DMSO-*d*_6_) δ 0.70 (s, 3H), 0.83–0.87 (m, 1H), 0.96 (s, 3H), 1.01 (s, 3H), 1.10 (s, 3H), 1.24–1.31 (m, 2H), 1.33 (s, 3H), 1.34–1.40 (m, 2H), 1.44 (d, *J* = 7.9 Hz, 1H), 1.50–1.60 (m, 3H), 1.62–1.74 (m, 2H), 1.77 (s, 3H), 1.98–2.08 (m, 3H), 2.33 (d, *J* = 15.4 Hz, 1H), 4.82 (d, *J* = 6.1 Hz, 1H), 6.06 (d, *J* = 6.4 Hz, 1H), 6.43 (s, 1H), 6.73 (s, 1H), 7.03 (dd, *J* = 8.5 and 1.9 Hz, 1H), 7.31 (d, *J* = 8.6 Hz, 1H), 7.60 (s, 1H), 7.87 (s, 1H), 8.96 (s, 1H), 10.83 (s, 1H), 12.09 (br. s., 1H). ^13^C-NMR (DMSO-*d*_6_) δ 11.89, 18.47, 22.28, 29.15, 29.91, 30.37, 30.49, 30.60, 31.43, 31.82, 32.89, 34.93, 35.02, 35.56, 35.76, 36.77, 36.87, 37.81, 39.89, 43.58, 44.31, 108.83, 113.45, 118.50, 119.22, 121.13, 122.49, 123.33, 123.93, 125.94, 128.02, 135.12, 140.79, 141.45, 144.11, 147.10, 180.00. HRMS (ESI) calcd. for C_37_H_43_ClNO_4_^−^ [M^−^]: 600.2886; found: 600.2871.

*(2R,4aS,6aS,12bR,14aS,14bR)-8-(5-Bromo-1H-indol-3-yl)-10,11-Dihydroxy-2,4a,6a,9,12b,14a-hexamethyl-1,2,3,4,4a,5,6,6a,8,12b,13,14,14a,14b-tetradecahydropicene-2-carboxylic acid* (**3m**): CAS: 2022987-45-5. Starting from 100 mg of celastrol (**2a**), compound **3m** (132 mg, 92%) was obtained as a wine-red solid according to the abovementioned procedure. ^1^H-NMR (DMSO-*d*_6_) δ 0.70 (s, 3H), 0.83–0.87 (m, 1H), 0.96 (s, 3H), 1.01 (s, 3H), 1.10 (s, 3H), 1.23–1.31 (m, 2H), 1.33 (s, 3H), 1.35–1.39 (m, 2H), 1.44 (d, *J* = 8.1 Hz, 1H), 1.50–1.60 (m, 3H), 1.62–1.73 (m, 2H), 1.77 (s, 3H), 1.97–2.07 (m, 3H), 2.33 (d, *J* = 15.4 Hz, 1H), 4.82 (d, *J* = 6.1 Hz, 1H), 6.05 (d, *J* = 6.4 Hz, 1H), 6.41 (s, 1H), 6.73 (s, 1H), 7.14 (dd, *J* = 8.6 and 1.8 Hz, 1H), 7.27 (d, *J* = 8.6 Hz, 1H), 7.74 (s, 1H), 7.87 (s, 1H), 8.96 (s, 1H), 10.84 (s, 1H), 12.07 (br. s., 1H). ^13^C-NMR (DMSO-*d*_6_) δ 11.90, 18.46, 22.27, 29.16, 29.91, 30.38, 30.49, 30.60, 31.44, 31.83, 32.89, 34.92, 35.02, 35.58, 35.75, 36.77, 36.87, 37.81, 43.58, 44.31, 108.83, 111.36, 113.95, 119.16, 121.11, 121.52, 122.48, 123.65, 123.77, 125.94, 128.75, 135.33, 140.78, 141.46, 144.12, 147.10, 180.00. HRMS (ESI) calcd. for C_37_H_43_BrNO_4_^−^ [M^−^]: 644.2381; found: 644.2367.

*(2R,4aS,6aS,12bR,14aS,14bR)-8-(5-Cyano-1H-indol-3-yl)-10,11-Dihydroxy-2,4a,6a,9,12b,14a-hexamethyl-1,2,3,4,4a,5,6,6a,8,12b,13,14,14a,14b-tetradecahydropicene-2-carboxylic acid* (**3n**): Starting from 100 mg of celastrol (**2a**), compound **3n** (26.9 mg, 20%) was obtained as a wine-red solid according to the above-mentioned procedure. ^1^H-NMR (DMSO-*d*_6_) δ 0.71 (s, 3H), 0.83–0.88 (m, 1H), 0.94 (s, 3H), 1.00 (s, 3H), 1.10 (s, 3H), 1.21–1.29 (m, 2H), 1.32 (s, 3H), 1.34–1.40 (m, 2H), 1.44 (d, *J* = 8.1 Hz, 1H), 1.50–1.61 (m, 3H), 1.63–1.74 (m, 2H), 1.78 (s, 3H), 2.01–2.09 (m, 3H), 2.33 (d, *J* = 15.4 Hz, 1H), 4.90 (d, *J* = 6.1 Hz, 1H), 6.11 (d, *J* = 6.4 Hz, 1H), 6.56 (s, 1H), 6.75 (s, 1H), 7.38 (dd, *J* = 8.4 and 1.3 Hz, 1H), 7.47 (d, *J* = 8.4 Hz, 1H), 7.91 (br. s., 1H), 8.12 (s, 1H), 9.01 (br. s., 1H), 11.24 (s, 1H), 12.08 (br. s., 1H). ^13^C-NMR (DMSO-*d*_6_) δ 11.89, 18.49, 22.26, 29.12, 29.91, 30.35, 30.49, 30.60, 31.43, 31.81, 32.88, 34.91, 35.56, 35.75, 36.79, 36.87, 37.80, 39.89, 43.62, 44.30, 100.70, 108.91, 113.25, 120.64, 121.08, 121.51, 122.42, 123.94, 124.71, 125.18, 125.63, 126.71, 138.36, 140.85, 141.53, 144.23, 147.51, 180.00. HRMS (ESI) calcd for C_38_H_43_N_2_O_4_^−^ [M^−^]: 591.3228; found: 591.3220.

*(2R,4aS,6aS,12bR,14aS,14bR)-8-(6-Fluoro-1H-indol-3-yl)-10,11-Dihydroxy-2,4a,6a,9,12b,14a-hexamethyl-1,2,3,4,4a,5,6,6a,8,12b,13,14,14a,14b-tetradecahydropicene-2-carboxylic acid* (**3o**): CAS: 2022987-47-7. Starting from 100 mg of celastrol (**2a**), compound **3o** (123.2 mg, 95%) was obtained as a wine-red solid according to the abovementioned procedure. ^1^H-NMR (DMSO-*d*_6_) δ 0.71 (s, 3H), 0.84–0.87 (m, 1H), 0.97 (s, 3H), 1.01 (s, 3H), 1.11 (s, 3H), 1.22–1.30 (m, 2H), 1.33 (s, 3H), 1.35–1.41 (m, 2H), 1.45 (d, *J* = 8.1 Hz, 1H), 1.51–1.61 (m, 3H), 1.63–1.74 (m, 2H), 1.79 (s, 3H), 1.96–2.08 (m, 3H), 2.34 (d, *J* = 15.4 Hz, 1H), 4.80 (d, *J* = 6.1 Hz, 1H), 6.10 (d, *J* = 6.4 Hz, 1H), 6.45 (s, 1H), 6.74 (s, 1H), 6.88 (td, *J* = 9.1 and 2.5 Hz, 1H), 7.25–7.34 (m, 2H), 7.88 (br. s., 1H), 9.00 (br. s., 1H), 10.73 (s, 1H), 12.05 (br. s., 1H). ^13^C-NMR (DMSO-*d*_6_) δ 11.87, 18.47, 22.31, 29.12, 29.91, 30.36, 30.49, 30.60, 31.43, 31.81, 32.88, 34.93, 35.12, 35.53, 35.74, 36.76, 36.89, 37.79, 43.57, 44.31, 103.95 (d, *J* = 23.1 Hz), 108.84, 109.24 (d, *J* = 23.1 Hz), 112.77 (d, *J* = 9.9 Hz), 119.43 (d, *J* = 5.5 Hz), 121.17, 122.55, 124.22, 125.99, 127.04 (d, *J* = 9.9 Hz), 133.33, 140.84, 141.42, 144.07, 147.03, 156.96 (d, *J* = 231.1 Hz), 180.01. HRMS (ESI) calcd. for C_37_H_43_FNO_4_^−^ [M^−^]: 584.3182; found: 584.3169.

*(2R,4aS,6aS,12bR,14aS,14bR)-8-(6-Chloro-1H-indol-3-yl)-10,11-Dihydroxy-2,4a,6a,9,12b,14a-hexamethyl-1,2,3,4,4a,5,6,6a,8,12b,13,14,14a,14b-tetradecahydropicene-2-carboxylic acid* (**3p**): CAS: 2022987-48-8. Starting from 100 mg of celastrol (**2a**), compound **3p** (129.2 mg, 97%) was obtained as a wine-red solid according to the abovementioned procedure. ^1^H-NMR (DMSO-*d*_6_) δ 0.70 (s, 3H), 0.85 (m, 1H), 0.94 (s, 3H), 1.00 (s, 3H), 1.09 (s, 3H), 1.21–1.28 (m, 2H), 1.30 (s, 3H), 1.32–1.39 (m, 2H), 1.43 (d, *J* = 8.1 Hz, 1H), 1.48–1.60 (m, 3H) 1.61–1.74 (m, 2H), 1.78 (s, 3H), 1.95–2.07 (m, 3H), 2.32 (d, *J* = 15.4 Hz, 1H), 4.81 (d, *J* = 5.9 Hz, 1H), 6.10 (d, *J* = 6.4 Hz, 1H), 6.36 (s, 1H), 6.72 (s, 1H), 6.96 (dd, *J* = 8.4, 1.8 Hz, 1H), 7.34 (d, *J* = 2.0 Hz, 1H), 7.58 (s, 1H), 7.88 (br. s., 1H), 8.99 (br. s., 1H), 10.75 (s, 1H), 12.05 (br. s., 1H). ^1^C-NMR (DMSO-*d*_6_) δ 11.86, 18.48, 22.31, 29.11, 29.90, 30.34, 30.49, 30.60, 31.43, 31.81, 32.88, 34.92, 35.11, 35.50, 35.69, 36.77, 36.87, 37.77, 43.58, 44.30, 108.85, 111.54, 118.95, 119.56, 120.66, 121.13, 122.44, 123.15, 125.75, 125.92, 125.99, 137.07, 140.84, 141.44, 144.08, 147.30, 180.01. HRMS (ESI) calcd. for C_37_H_43_ClNO_4_^−^ [M^−^]: 600.2886; found: 600.2879.

*Ethyl (2R,4aS,6aS,12bR,14aS,14bR)-10,11-dihydroxy-8-(1H-indol-3-yl)-2,4a,6a,9,12b,14a-hexamethyl-1,2,3,4,4a,5,6,6a,8,12b,13,14,14a,14b-tetradecahydropicene-2-carboxylate* (**1a2b**): Starting from 150 mg **2b**, compound **1a2b** (165.9 mg, 89%) was obtained as a wine-red solid. ^1^H-NMR (DMSO-*d*_6_) δ 0.58 (s, 3H), 0.83–0.88 (m, 1H), 0.97 (s, 3H), 1.02 (s, 3H), 1.11 (s, 3H), 1.13–1.15 (m, 2H), 1.15–1.17 (m, 1H) 1.17–1.19 (m, 1H), 1.29–1.35 (m, 1H), 1.36 (s, 3H), 1.37–1.41 (m, 1H), 1.46 (d, *J* = 7.9 Hz, 1H), 1.56 (td, *J* = 13.4 and 3.9 Hz, 2H), 1.60–1.66 (m, 2H), 1.72 (td, *J* = 14.0 and 5.9 Hz, 1H), 1.80 (s, 3H), 1.95–2.03 (m, 2H), 2.08 (d, *J* = 12.5 Hz, 2H), 2.36 (d, *J* = 15.6 Hz, 1H), 3.87–3.98 (m, 2H), 4.83 (d, *J* = 6.1 Hz, 1H), 6.13 (d, *J* = 6.4 Hz, 1H), 6.31 (s, 1H), 6.73 (s, 1H), 6.96 (t, *J* = 7.4 Hz, 1H), 7.05 (t, *J* = 7.5 Hz, 1H), 7.31 (d, *J* = 8.1 Hz, 1H), 7.61 (d, *J* = 8.1 Hz, 1H), 7.85 (s, 1H), 8.94 (s, 1H), 10.61 (s, 1H). ^13^C-NMR (DMSO-*d*_6_) δ 11.42, 14.10, 17.81, 20.77, 21.68, 28.68, 29.39, 30.01, 30.05, 30.17, 31.34, 32.44, 34.32, 34.81, 35.05, 35.30, 36.32, 36.36, 37.31, 43.07, 43.80, 59.83, 108.41, 111.50, 118.22, 118.74, 118.82, 120.72, 121.49, 122.31, 125.74, 126.58, 136.27, 140.33, 141.03, 143.59, 146.19, 177.43. HRMS (ESI) calcd. for C_39_H_48_NO_4_^−^ [M^−^]: 594.3589; found: 594.3578.

*Methyl (2R,4aS,6aS,12bR,14aS,14bR)-10-(butanoyloxy)-11-hydroxy-8-(1H-indol-3-yl)-2,4a,6a,9,12b,14a-hexamethyl-1,2,3,4,4a,5,6,6a,8,12b,13,14,14a,14b-tetradecahydropicene-2-carboxylate* (**1a2c**): Starting from 68.9 mg **2c**, compound **1a2c** (24.5 mg, 29%) was obtained as a pink solid according to the above-mentioned procedure. ^1^H-NMR (DMSO-*d*_6_) δ 0.55 (br. s., 3H), 0.89 (d, *J* = 11.74 Hz, 1H), 0.92–0.96 (m, 3H), 0.97 (br. s., 3H), 1.02 (br. s., 3H), 1.11 (br. s., 3H), 1.35 (br. s., 3H), 1.37–1.41 (m, 2H), 1.46 (d, *J* = 6.42 Hz, 1H), 1.49–1.60 (m, 4H), 1.63 (dd, *J* = 14.21, 7.06 Hz, 4H), 1.72 (br. s., 3H), 1.82 (dd, *J* = 15.96, 11.55 Hz, 1H), 1.98 (t, *J* = 12.29 Hz, 1H), 2.03–2.07 (m, 1H), 2.10 (br. s., 1H), 2.32 (d, *J* = 15.22 Hz, 1H), 2.53 (t, *J* = 7.20 Hz, 2H), 3.49 (br. s., 3H), 4.86 (d, *J* = 5.50 Hz, 1H), 6.18 (d, *J* = 5.69 Hz, 1H), 6.29 (br. s., 1H), 6.85 (br. s., 1H), 6.91–6.99 (m, 1H), 7.05 (t, *J* = 7.06 Hz, 1H), 7.30 (d, *J* = 7.70 Hz, 1H), 7.61 (d, *J* = 7.89 Hz, 1H), 9.25 (br. s., 1H), 10.65 (br. s., 1H). ^13^C-NMR (DMSO-*d*_6_) δ 11.77, 13.47,17.79, 18.05, 21.79, 28.65, 29.48, 30.00 , 30.15, 30.18, 31.34, 32.35, 34.37, 34.71, 34.89, 35.08, 35.13, 36.35, 36.75, 37.21, 39.85, 43.16, 43.80, 51.47, 109.84, 111.55, 118.25, 118.34, 118.85, 120.89, 121.42, 122.11, 125.80, 126.45, 127.44, 135.40, 136.32, 146.09, 147.30, 147.34, 171.09, 178.01.

## 4. Conclusions

In conclusion, an experimentally simple, highly efficient Michael-type Friedel-Crafts addition of indoles to the *para*-quinone methide of celastrol was developed for the synthesis of celastrol derivatives. A diverse set of indoles could react with celastrol to produce the desired products in moderate to good yields (20–99%) with only 5 mol % of AlCl_3_·6H_2_O as catalyst in 3 h of reaction time. This method could be used extensively for the synthesis of C6-indole substituted celastrols.

## Figures and Tables

**Scheme 5:**
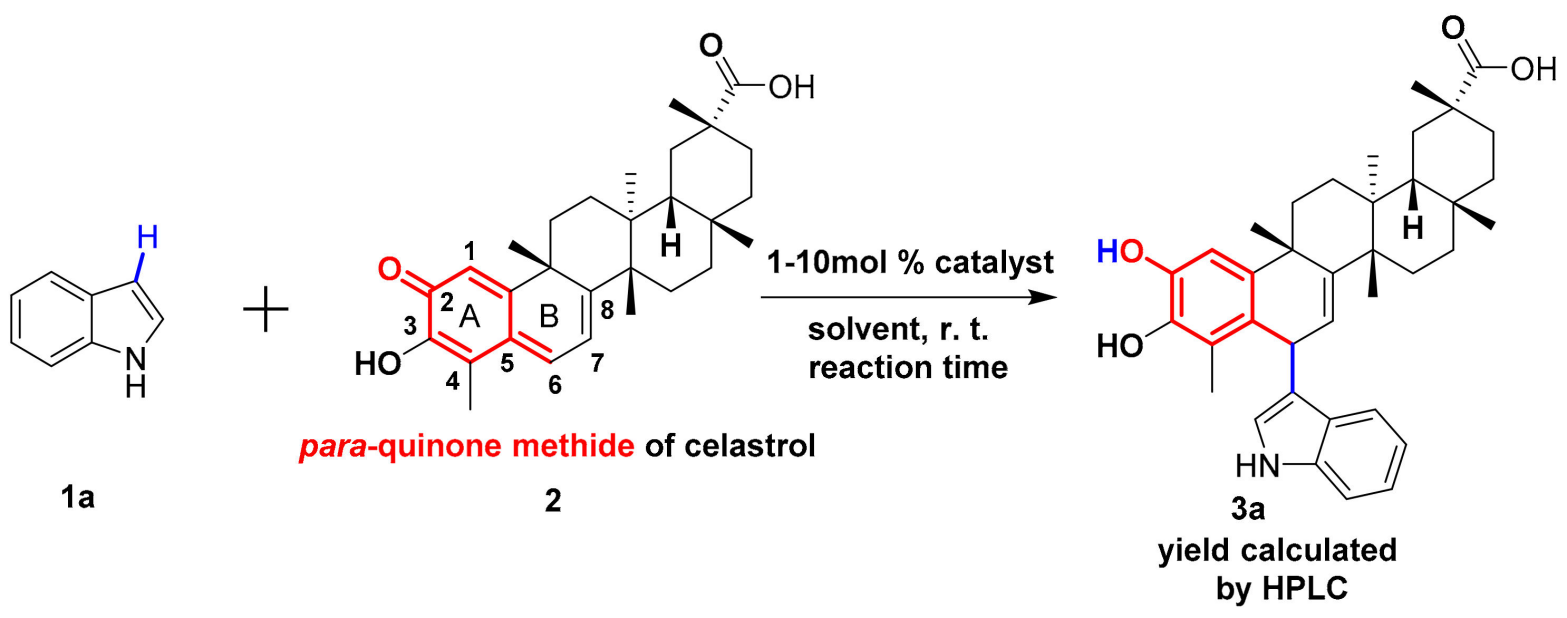
Synthesis of compound 3a.

**Table 1:**
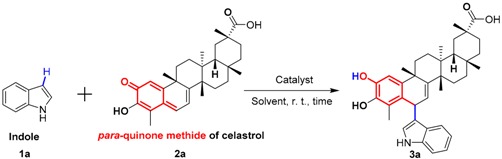
The screening of metal catalysts for the Friedel-Crafts alkylation of indoles with celastrol ^a^.

Entry	Catalyst (mol %)	Solvent (1 mL)	Indole 1a (equiv.)	Time (h)	Yield of 3a ^b^ (%)
1	/	DCM	2	12	2
2	Pd[P(C_6_H_5_)_3_]_4_ (10)	DCM	2	12	12
3	Pd(OAc)_2_ (10)	DCM	2	12	28
4	FePO_4_·6H_2_O (10)	DCM	2	12	9
5	Al(OH)_3_ (10)	DCM	2	12	0
6	Al_2_(SO_4_)_3_·18H_2_O (10)	DCM	2	12	12
7	MgCl_2_·6H_2_O (10)	DCM	2	12	64
8	ZnCl_2_ (10)	DCM	2	12	80
9	FeCl_3_·6H_2_O (10)	DCM	2	12	93
10	AlCl_3_·6H_2_O (10)	DCM	2	12	99
11	AlCl_3_·6H_2_O (10)	MeOH	2	12	95
12	AlCl_3_·6H_2_O (10)	THF	2	12	87
13	AlCl_3_·6H_2_O (10)	Dioxane	2	12	84
14	AlCl_3_·6H_2_O (10)	ACN	2	12	90
15	AlCl_3_·6H_2_O (10)	DMF	2	12	93
16	AlCl_3_·6H_2_O (10)	DCM	2	6	99
17	AlCl_3_·6H_2_O (10)	DCM	2	3	99
18	AlCl_3_·6H_2_O (10)	DCM	2	1.5	68
19	AlCl_3_·6H_2_O (10)	DCM	2	0.5	53
20	AlCl_3_·6H_2_O (10)	DCM	1.5	3	100
21	AlCl_3_·6H_2_O (10)	DCM	1.2	3	99 ^c^
22	AlCl_3_·6H_2_O (10)	DCM	1	3	98
23	AlCl_3_·6H_2_O (5)	DCM	1.2	3	99 ^d^
24	AlCl_3_·6H_2_O (1)	DCM	1.2	3	98 ^e^

^a^ *Reaction conditions*: **1a** (1–2 equiv., 0.045–0.090 mmol), **2a** (0.045 mmol, 20 mg), and metal catalyst (1–10 mol %) were dissolved in DCM (1 mL) at room temperature; ^b^ Yield was calculated by HPLC, see Materials and Methods; ^c^ Conditions: **1a** (1.2 equiv.), **2a** (50 mg), and AlCl_3_·6H_2_O (10 mol %) in DCM at room temperature for 3 h; ^d^ Conditions: **1a** (1.2 equiv.), **2a** (100 mg), and AlCl_3_·6H_2_O (5 mol %) in DCM at room temperature for 3 h; ^e^ Conditions: **1a** (1.2 equiv.), **2a** (250 mg), and AlCl_3_·6H_2_O (1 mol %) in DCM at room temperature for 3 h. Abbreviations: equiv = equivalent, DCM = methylene chloride, THF = tetrahydrofuran, ACN = acetonitrile, DMF = dimethylformamide, r.t. = room temperature.

## Supplementary Materials

Supplementary materials are available online.
