# Supplementary material for: AlCl3·6H2O-Catalyzed Friedel-Crafts Alkylation of Indoles by the para-Quinone Methide Moiety of Celastrol
Source: Molecules. 2017 May 16;22(5):742. doi: 10.3390/molecules22050742 (PMC6154665; doi:10.3390/molecules22050742)
Supplement: Supplementary file 1 [file molecules-22-00742-s001.pdf]

Supporting information for

$AlCl_3 \cdot 6H_2O$-Catalyzed Friedel–Crafts Alkylation of Indoles by *para*-Quinone Methide moiety of Celastrol

Yi Zhu[§a], Ziwen Chen[§a], Zhenfei Huang[a], Siwei Yan[a], Zhuoer Li[a], Hu Zhou[a,b], Xiaokun Zhang[a,b], Ying Su[⋆a,b] and Zhiping Zeng[⋆a]

a School of Pharmaceutical Science, Fujian Provincial Key laboratory of Innovative Drug Target Research, Xiamen University, Xiamen 361005, China;

b Sanford Burnham Prebys Medical Discovery Institute, 10901 N. Torrey Pines Road, La Jolla, CA 92037, USA

§*These authors contributed equally to this work.*

⋆To whom correspondence should be addressed:

Zhi-ping Zeng, Tel: +86 592 218 1851; Fax: +86 592 218 1879;

Email: zengzhiping@xmu.edu.cn

Ying Su, Tel: +86 592 218 1851; Fax: +86 592 218 1879;

Email: ysu@sbpdiscovery.org

Contents

1. Regioselectivity analysis of our reaction 1

2. HPLC method for the determination of reaction yield in screening 2

3. HPLC spectra and results of reaction optimization in Table 1... 6

4. NMR spectra of synthesized compounds 18

1. Regioselectivity analysis of our reaction

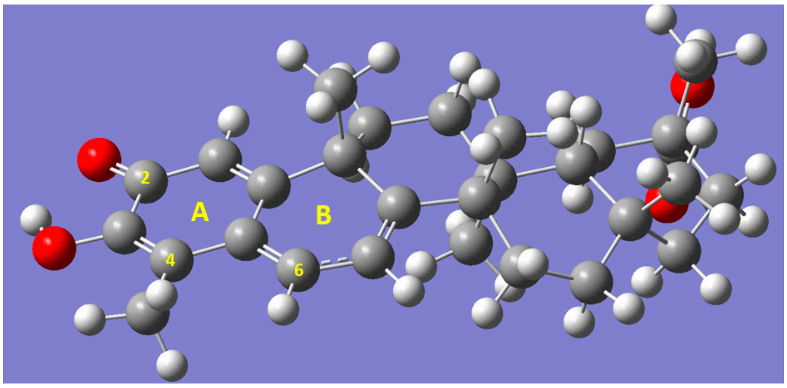

Figure S1. The 3D structure of celastrol

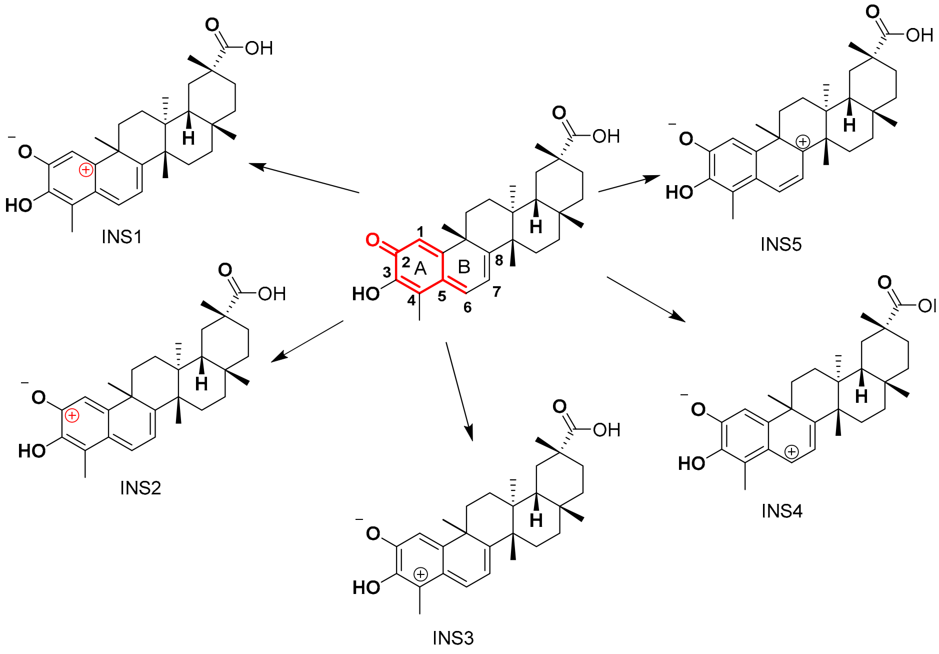

Scheme S1. The possible carbocationic intermediates produced by celastrol

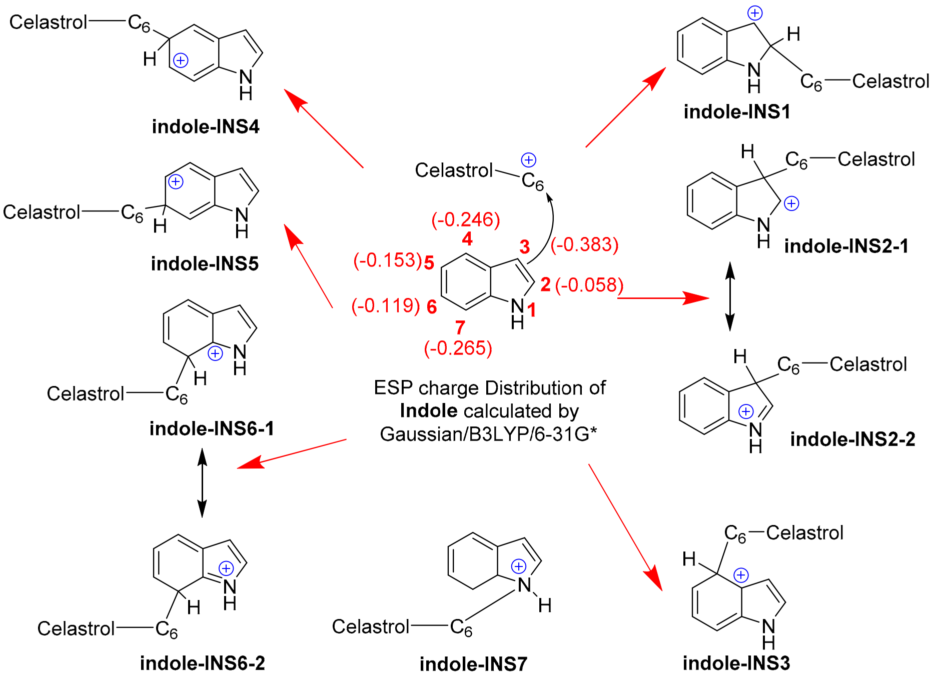

Scheme S2. The possible intermediates produced by indole

2. HPLC method for the determination of reaction yield in screening

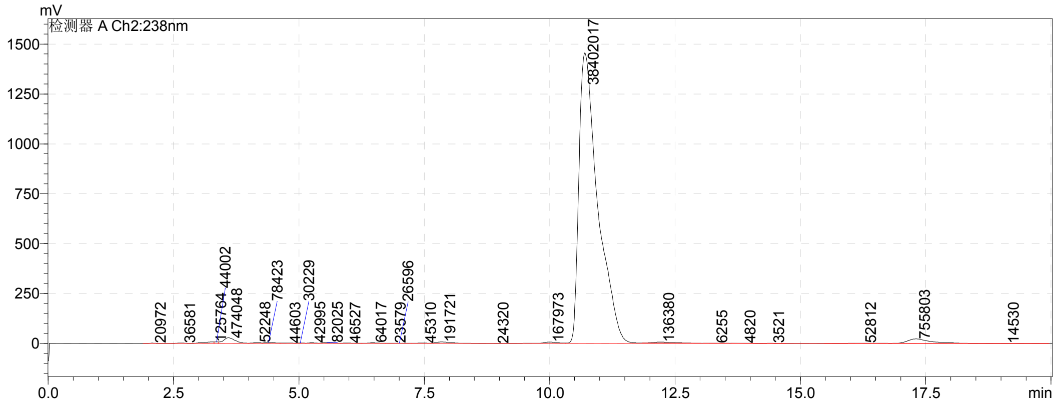

Figure S2. The HPLC spectrum of 2 mg/mL **3a**

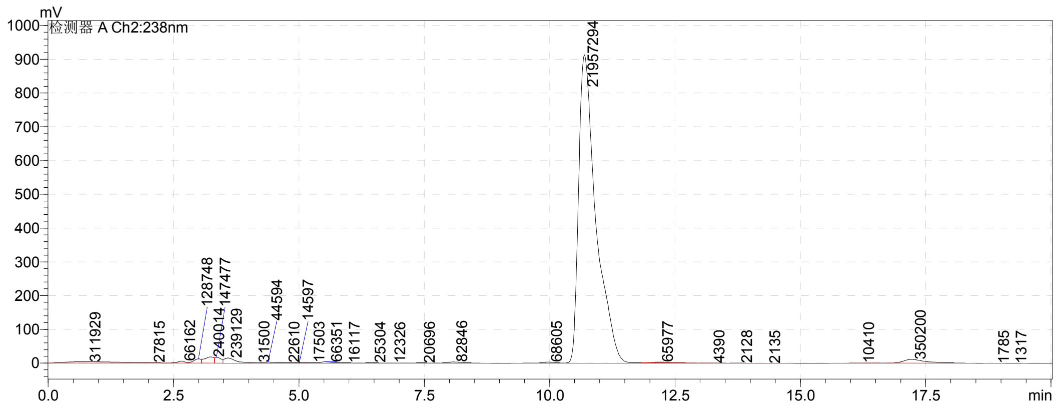

Figure S3. The HPLC spectrum of 1 mg/mL 3a

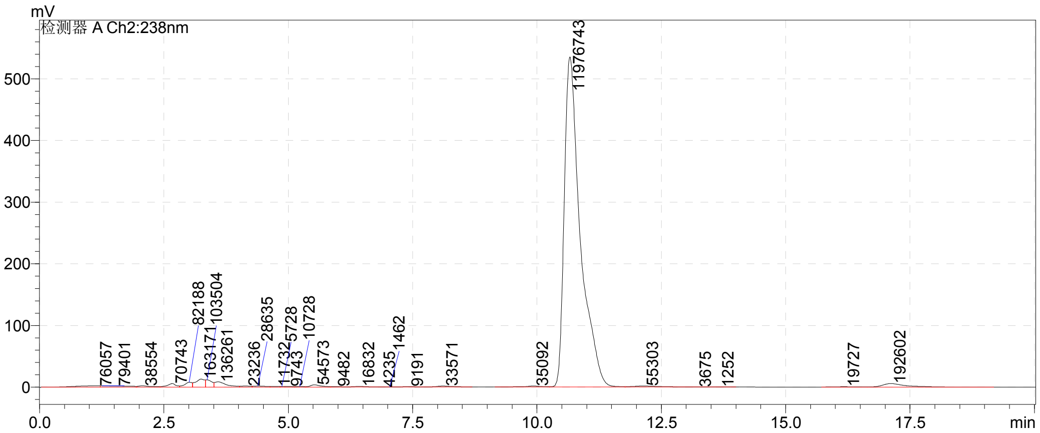

Figure S4. The HPLC spectrum of 0.5 mg/mL 3a

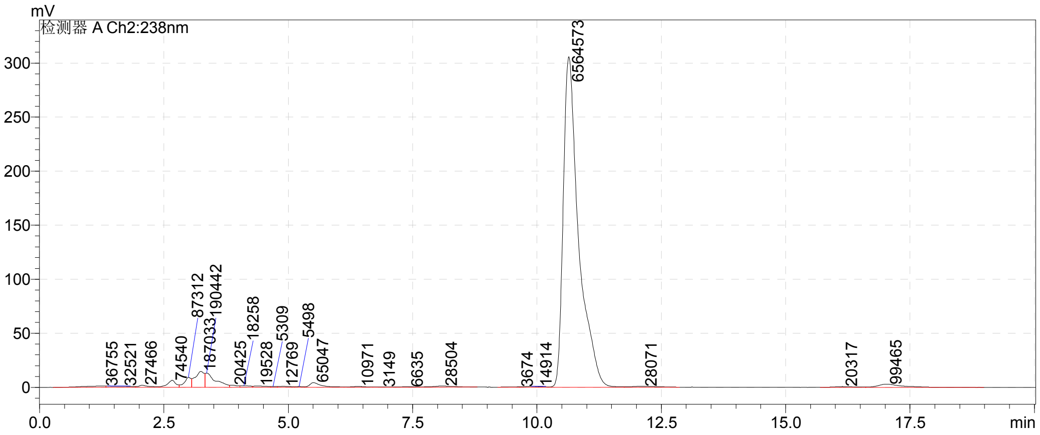

Figure S5. The HPLC spectrum of 0.25 mg/mL 3a

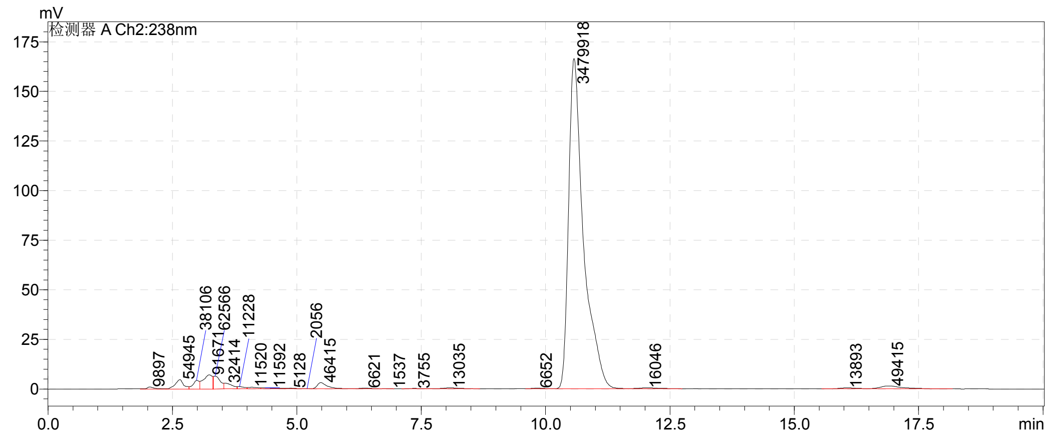

Figure S6. The HPLC spectrum of 0.125 mg/mL 3a

Table S1. The summarized HPLC results of **3a** with different concentrations

Entry	Retention (min)	Concentration (mg/mL)	Peak area
1	10.698	2	38402017
2	10.690	1	21957294
3	10.657	0.5	11976743
4	10.634	0.25	6564573
5	10.568	0.125	3479918

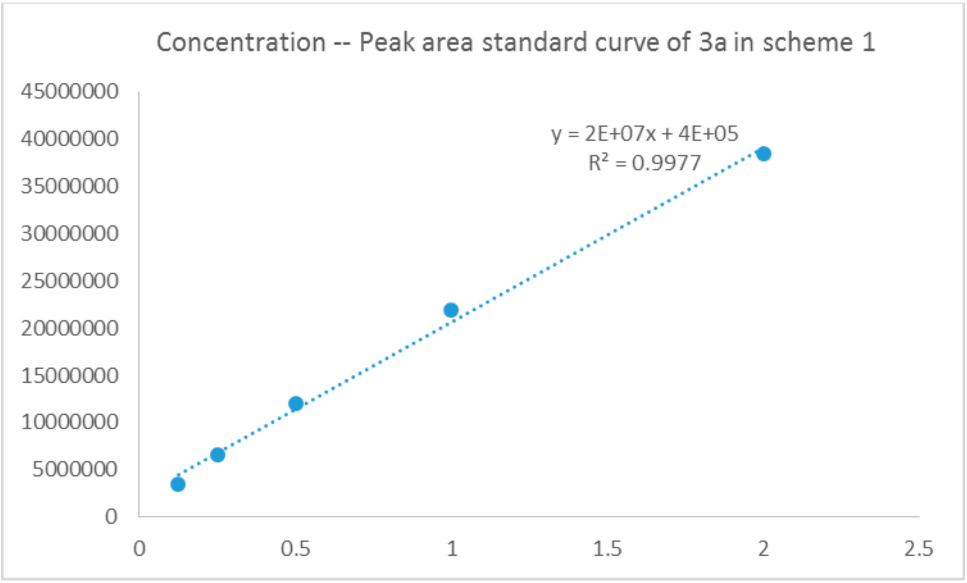

Figure S7. Concentration – Peak area standard curve of 3a

Fitting formula:

$y = 2E+07x + 4E+05$ $R^2 = 0.9977$

3. HPLC spectra and results of reaction optimization in Table 1

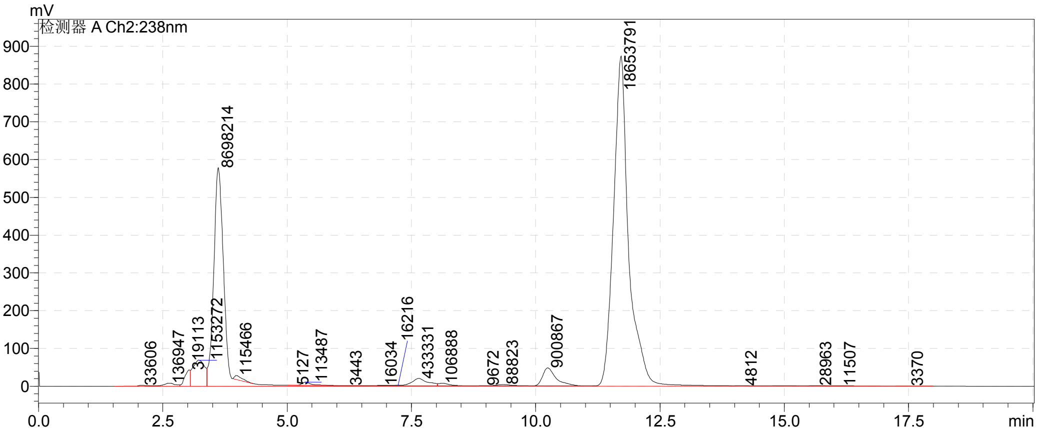

Figure S8 The HPLC spectrum of entry 1 in table 1

compound	Retention time	Peak area	Amount (mg)	Yield
2a	10.238	900867	0.6	**2 %**
1a	11.711	18653791		

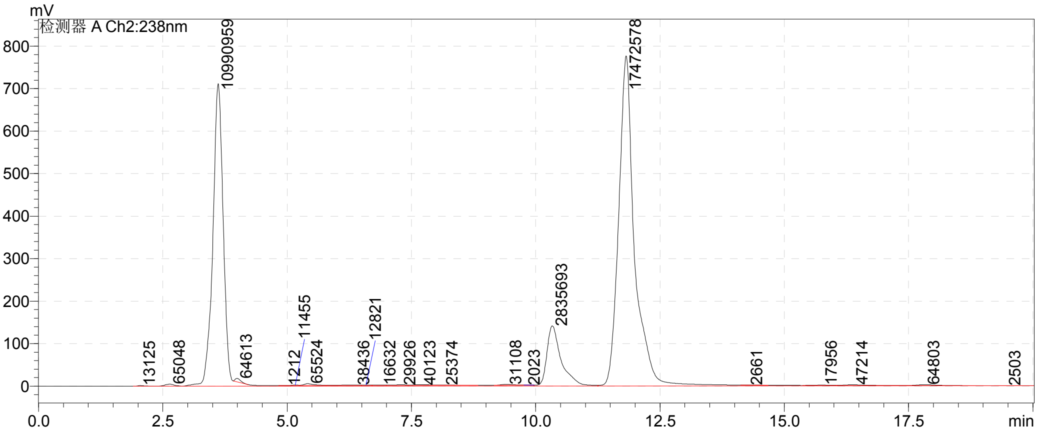

Figure S9 The HPLC spectrum of entry 2 in table 1

compound	Retention time	Peak area	Amount (mg)	Yield
2a	10.329	2835693	3.0	**12 %**
1a	11.815	17472578		

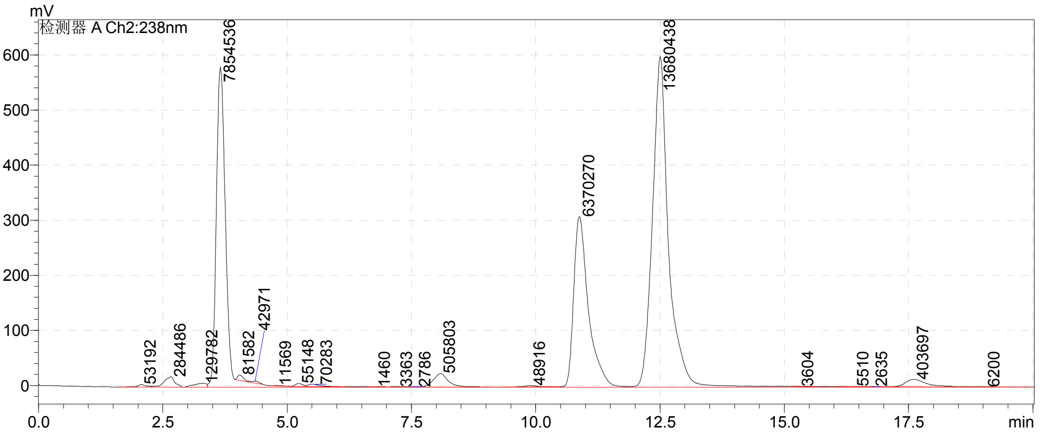

Figure S10 The HPLC spectrum of entry 3 in table 1

compound	Retention time	Peak area	Amount (mg)	Yield
2a	10.873	6370270	7.5	**28 %**
1a	12.501	13680438		

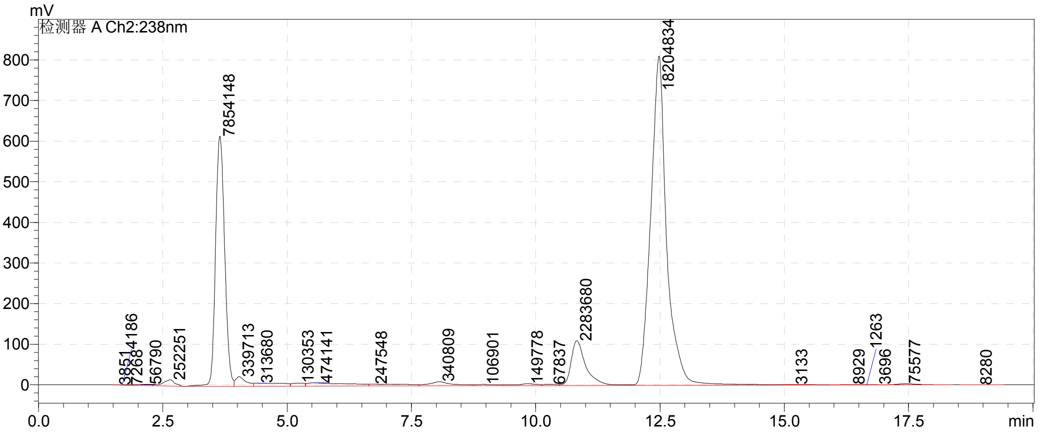

Figure S11 The HPLC spectrum of entry 4 in table 1

compound	Retention time	Peak area	Amount of (mg)	Yield
2a	1	2283680	2.3	**9 %**
1a	2	18204834		

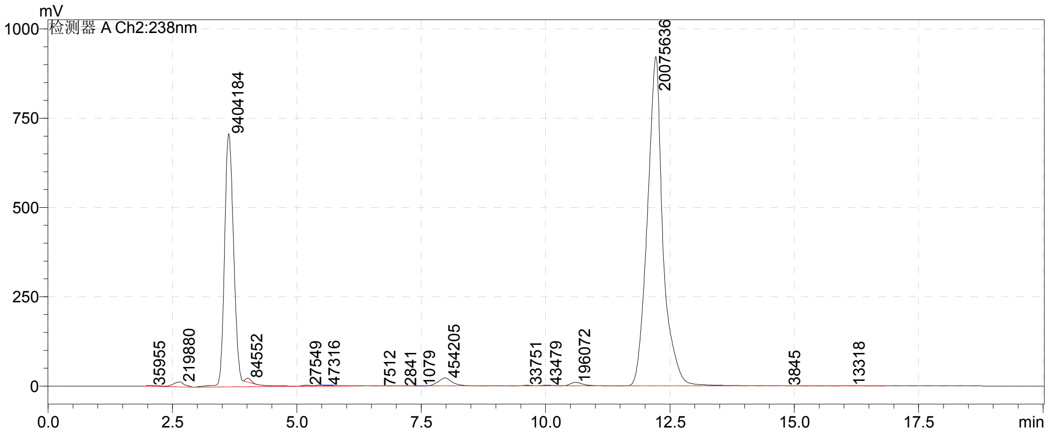

Figure S12 The HPLC spectrum of entry 5 in table 1

compound	Retention time	Peak area	Amount (mg)	Yield
2a	10.599	196072	0	**0 %**
1a	12.212	20075636		

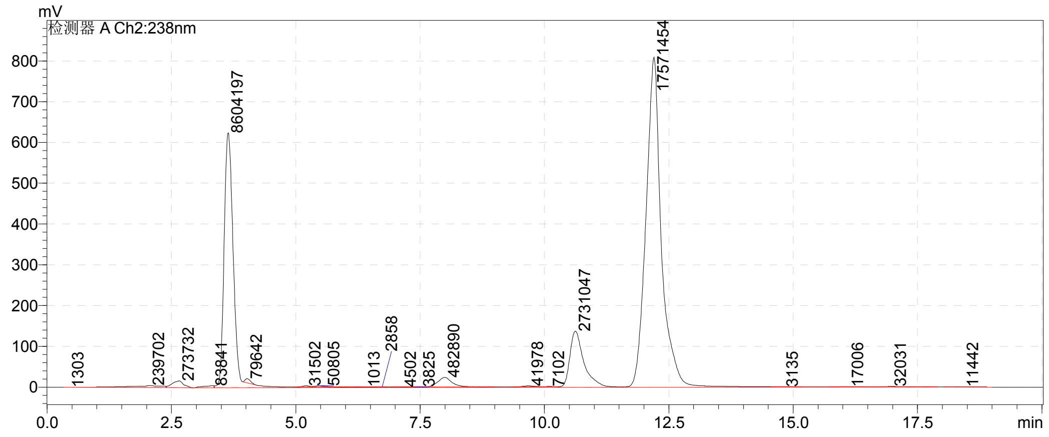

Figure S13 The HPLC spectrum of entry 6 in table 1

compound	Retention time	Peak area	Amount (mg)	Yield
2a	10.615	2731047	2.9	**11 %**
1a	12.197	17571454		

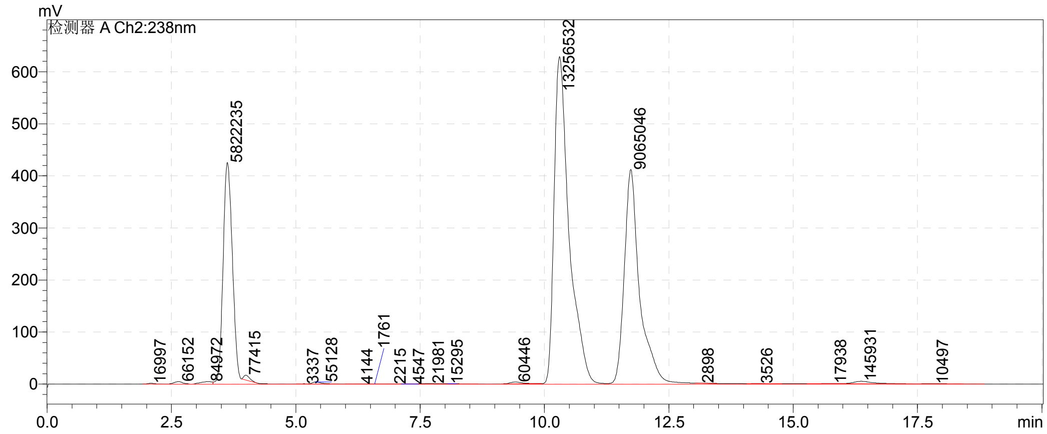

Figure S14 The HPLC spectrum of entry 7 in table 1

compound	Retention time	Peak area	Amount (mg)	Yield
2a	10.298	13256532	16.1	**64 %**
1a	11.731	9065046		

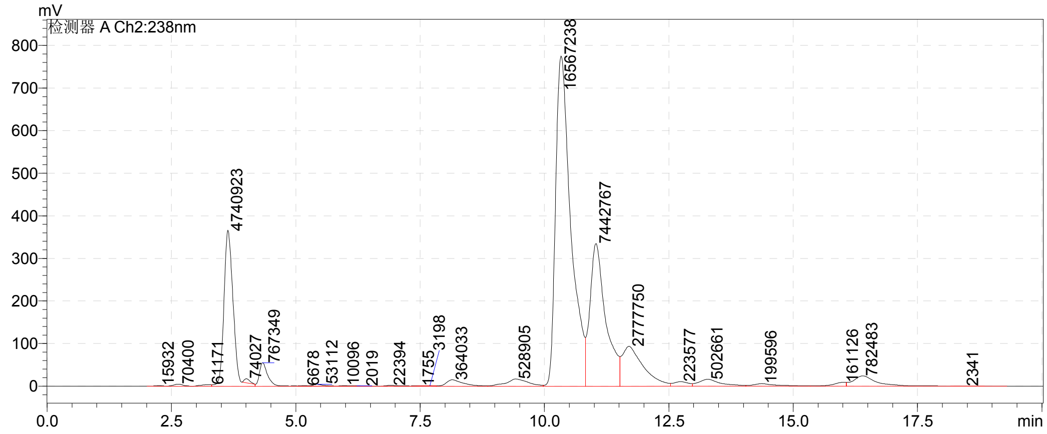

Figure S15 The HPLC spectrum of entry 8 in table 1

compound	Retention time	Peak area	Amount (mg)	Yield
2a	10.330	16567238	20.2	**80 %**
1a	11.694	2777750		

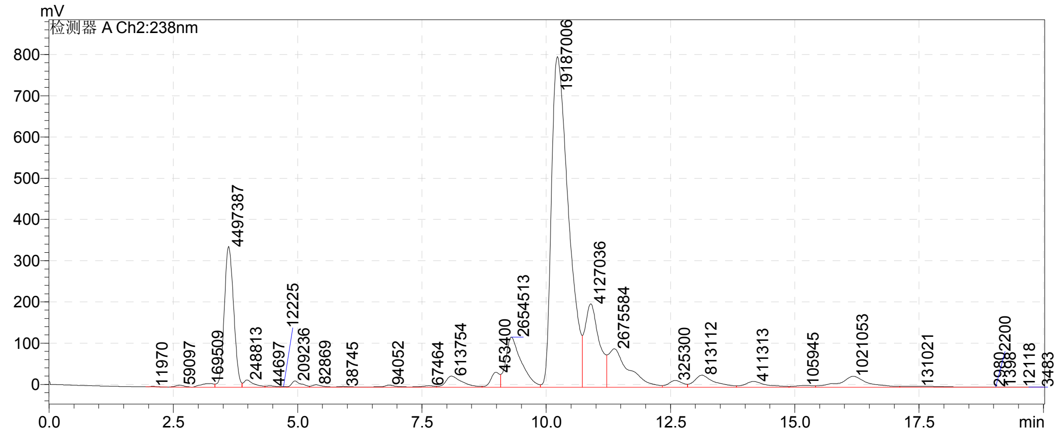

Figure S16 The HPLC spectrum of entry 9 in table 1

compound	Retention time	Peak area	Amount (mg)	Yield
2a	10.220	19187006	23.5	**93 %**
1a	11.365	2675584		

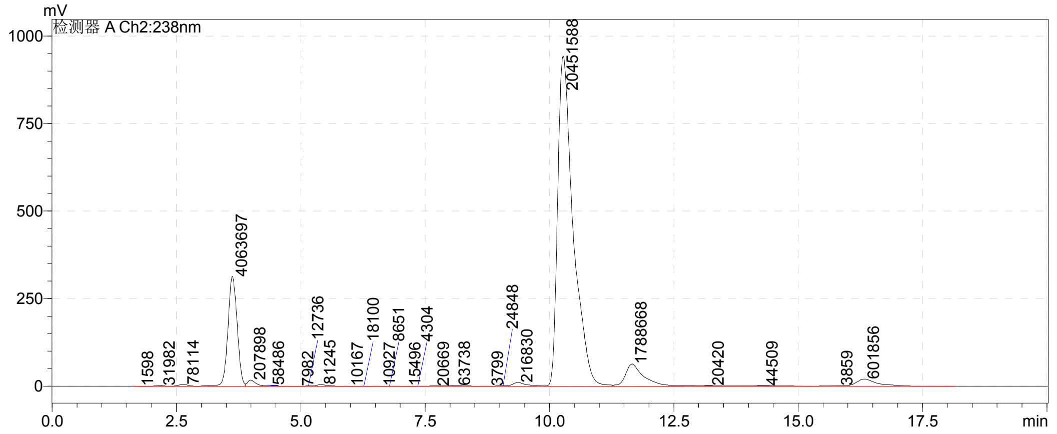

Figure S17 The HPLC spectrum of entry 10 in table 1

compound	Retention time	Peak area	Amount (mg)	Yield
2a	10.271	20451588	25.0	**99 %**
1a	11.655	1788668		

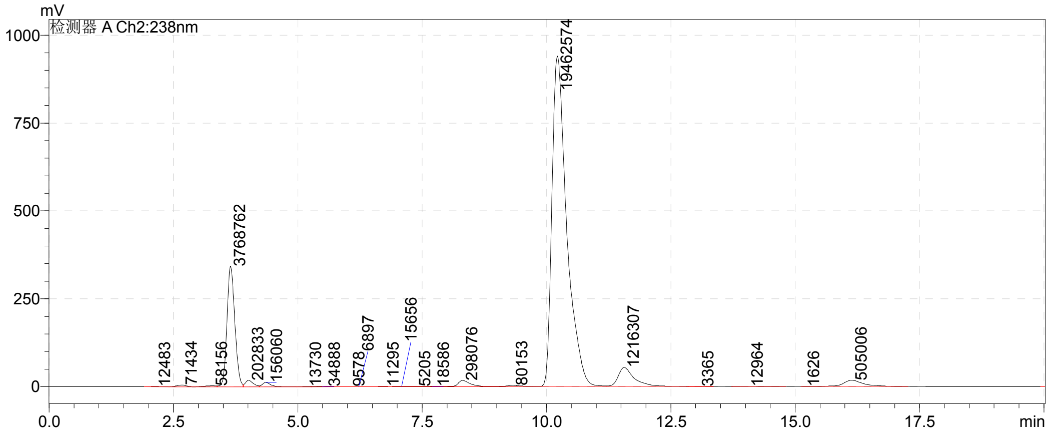

Figure S18 The HPLC spectrum of entry 11 in table 1

compound	Retention time	Peak area	Amount (mg)	Yield
2a	10.215	19462574	23.8	**94 %**
1a	11.558	1216307		

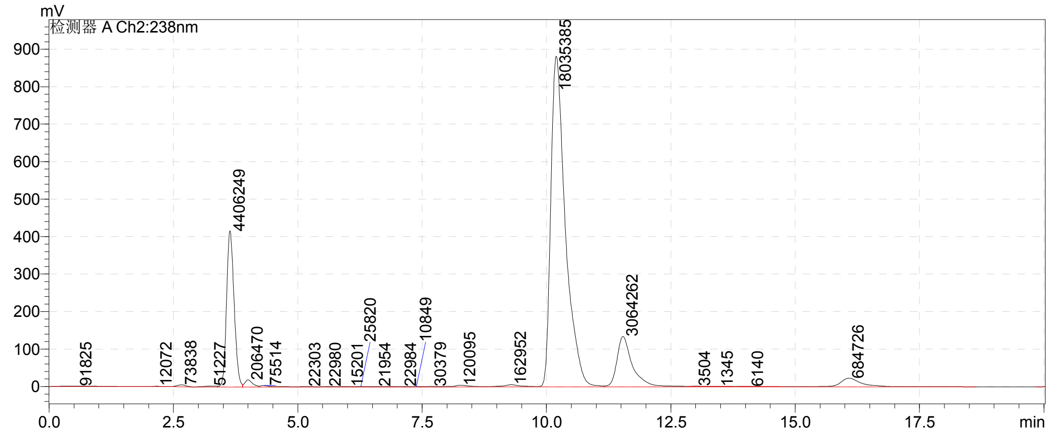

Figure S19 The HPLC spectrum of entry 12 in table 1

compound	Retention time	Peak area	Amount (mg)	Yield
2a	10.192	18035385	20.0	**87 %**
1a	11.533	3064262		

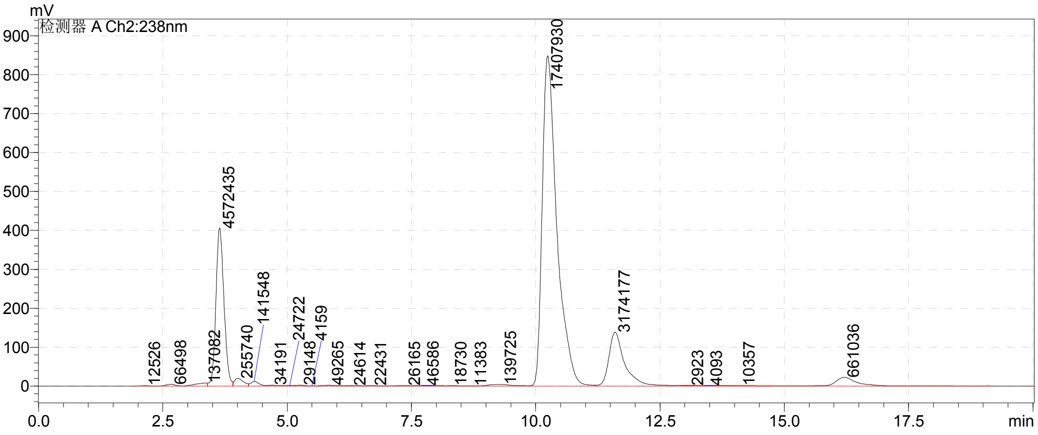

Figure S20 The HPLC spectrum of entry 13 in table 1

compound	Retention time	Peak area	Amount (mg)	Yield
2a	10.237	17407930	21.2	**84 %**
1a	11.590	3174177		

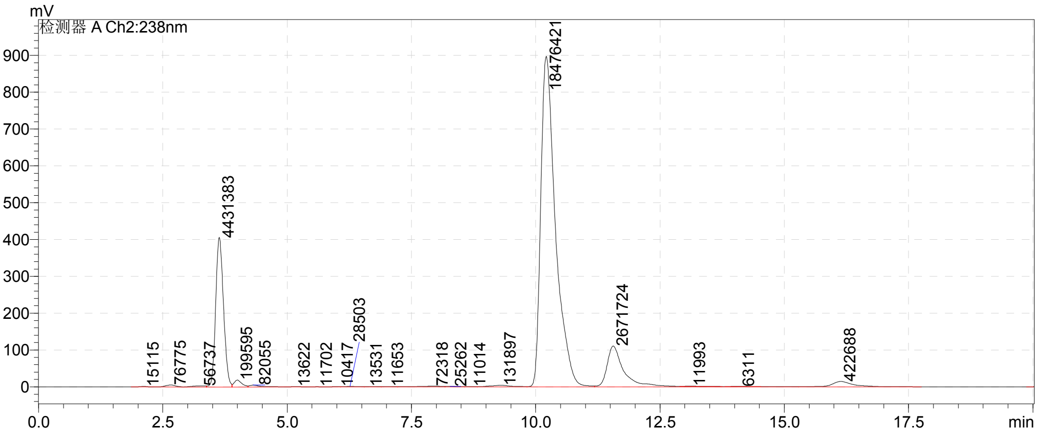

Figure S21 The HPLC spectrum of entry 14 in table 1

compound	Retention time	Peak area	Amount (mg)	Yield
2a	10.207	18476421	22.7	**90 %**
1a	11.554	2671724		

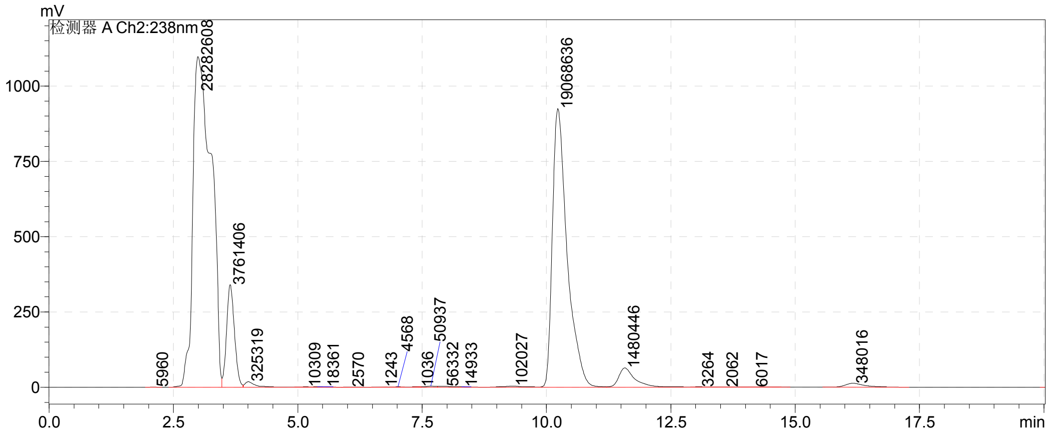

Figure S22 The HPLC spectrum of entry 15 in table 1

compound	Retention time	Peak area	Amount (mg)	Yield
2a	10.224	19068636	23.3	**92 %**
1a	11.570	3174177		

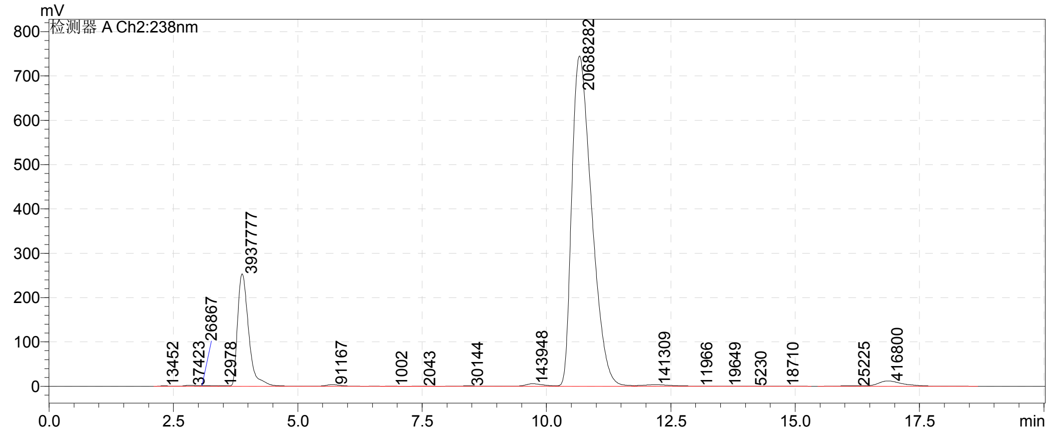

Figure S23 The HPLC spectrum of entry 16 in table 1

compound	Retention time	Peak area	Amount (mg)	Yield
2a	10.659	20688282	25.3	**99 %**
1a	12.189	141309		

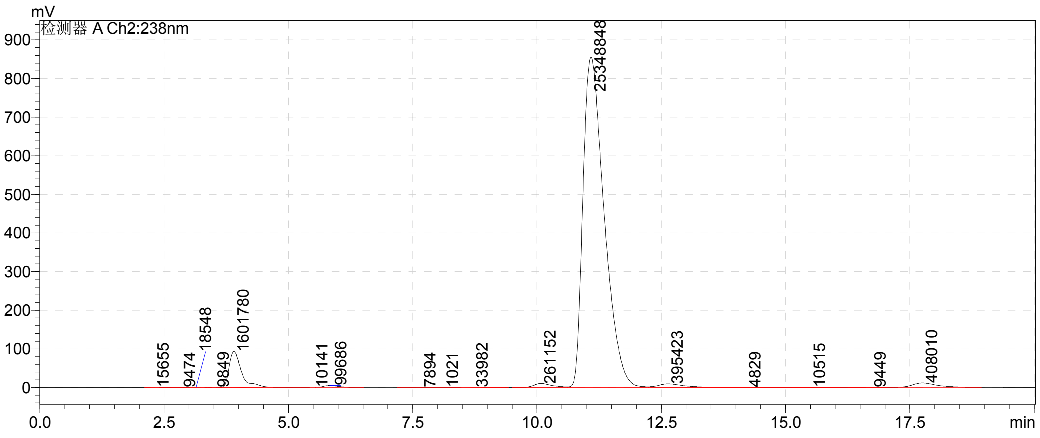

Figure S24 The HPLC spectrum of entry 17 in table 1

compound	Retention time	Peak area	Amount (mg)	Yield
2a	10.682	25348848	62.4	**99 %**
1a	12.234	395423		

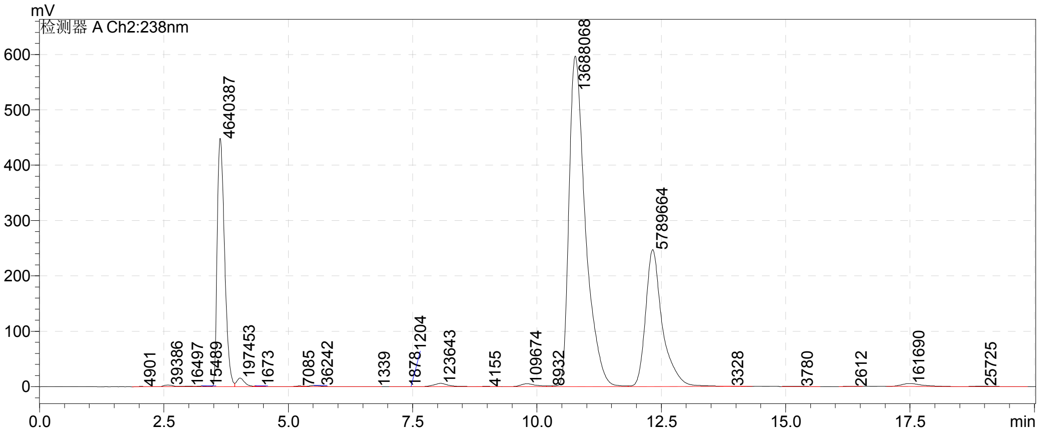

Figure S25 The HPLC spectrum of entry 18 in table 1

compound	Retention time	Peak area	Amount (mg)	Yield
2a	10.466	13688068	16.6	**66 %**
1a	12.022	5789664		

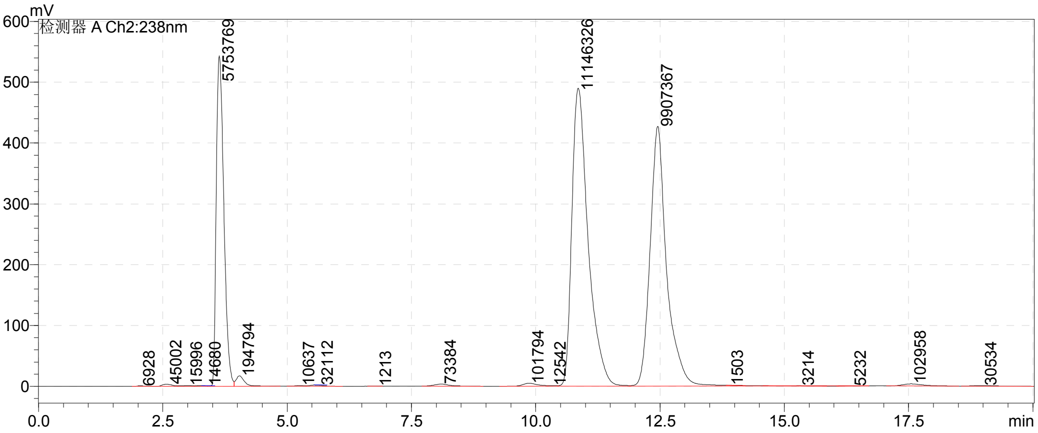

Figure S26 The HPLC spectrum of entry 19 in table 1

compound	Retention time	Peak area	Amount (mg)	Yield
2a	10.502	11146326	13.4	**53 %**
1a	12.150	9907367		

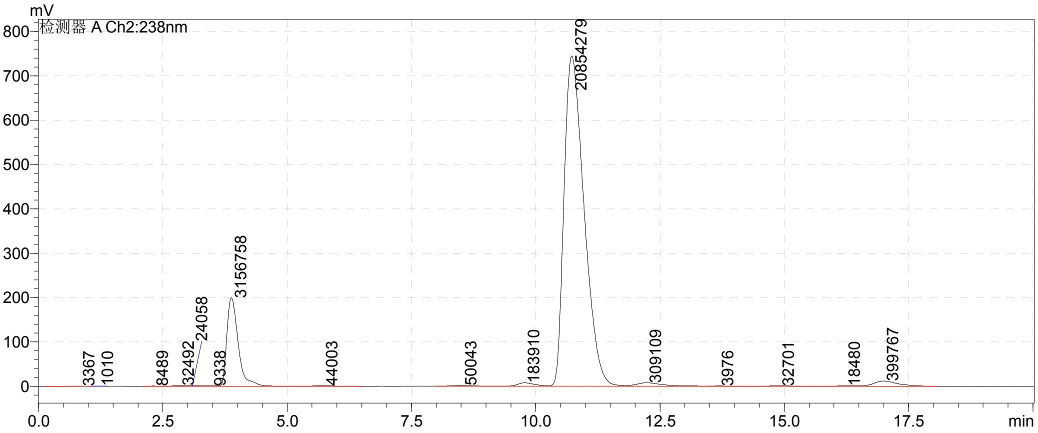

Figure S27 The HPLC spectrum of entry 20 in table 1

compound	Retention time	Peak area	Amount (mg)	Yield
2a	10.422	20854279	25.5	**100 %**
1a	12.022	309109		

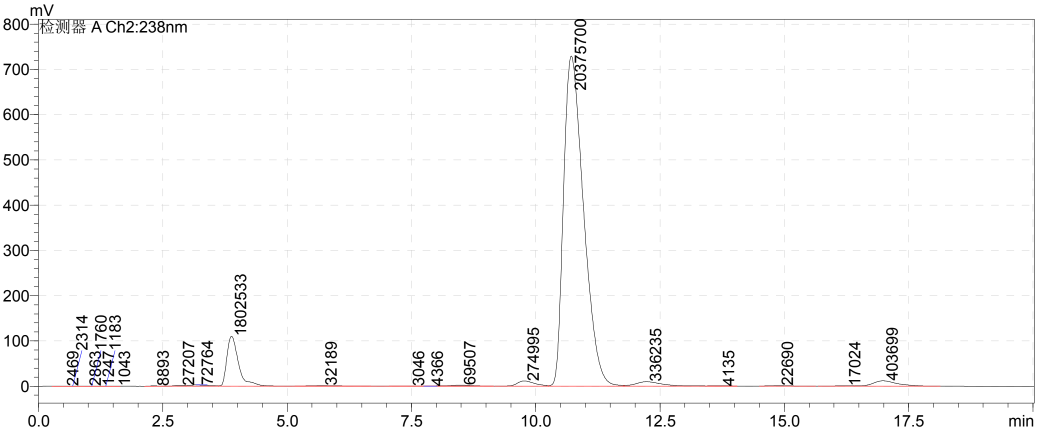

Figure S28 The HPLC spectrum of entry 21 in table 1

compound	Retention time	Peak area	Amount (mg)	Yield
2a	1	20375700	25.0	**99 %**
1a	2	336235		

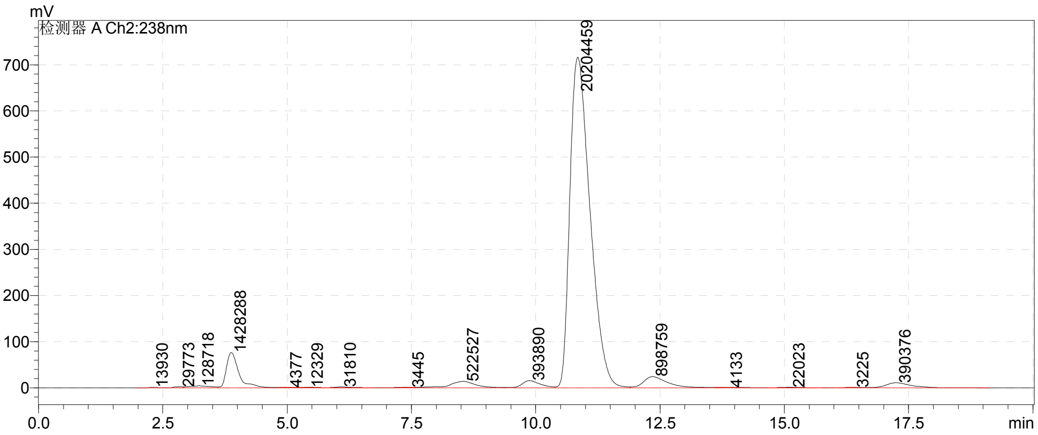

Figure S29 The HPLC spectrum of entry 22 in table 1

compound	Retention time	Peak area	Amount (mg)	Yield
2a	10.440	20204459	24.7	**98 %**
1a	12.045	898759		

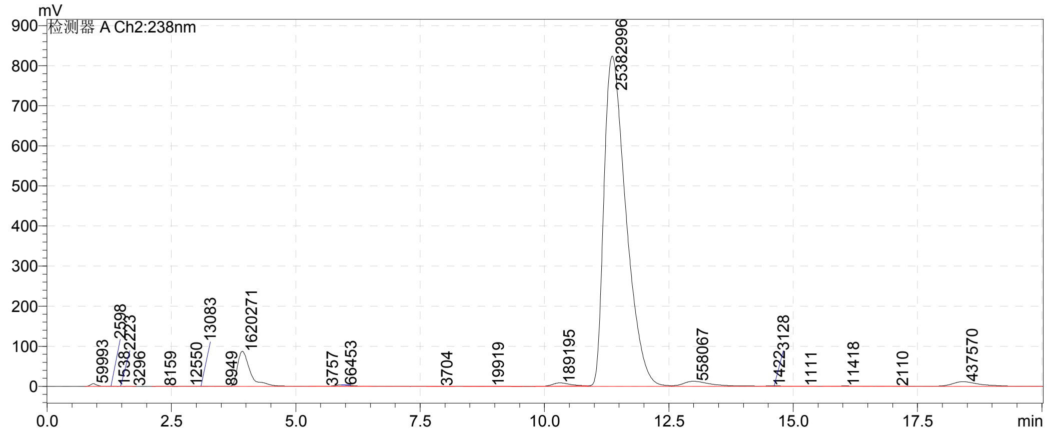

Figure S30 The HPLC spectrum of entry 23 in table 1

compound	Retention time	Peak area	Amount (mg)	Yield
2a	10.857	25382996	124.9	**99 %**
1a	12.290	558067		

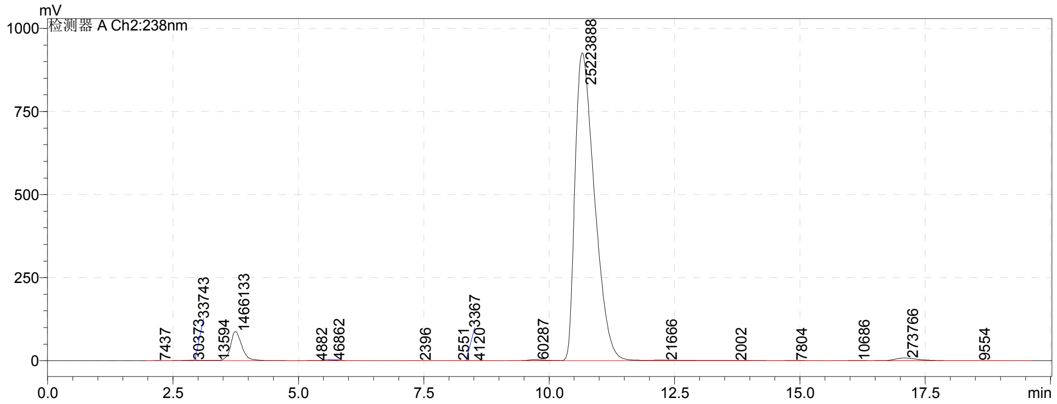

Figure S31 The HPLC spectrum of entry 24 in table 1

compound	Retention time	Peak area	Amount (mg)	Yield
2a	10.657	25223888	310.3	**98 %**
1a	12.270	21666		

4. NMR spectra of synthesized compounds

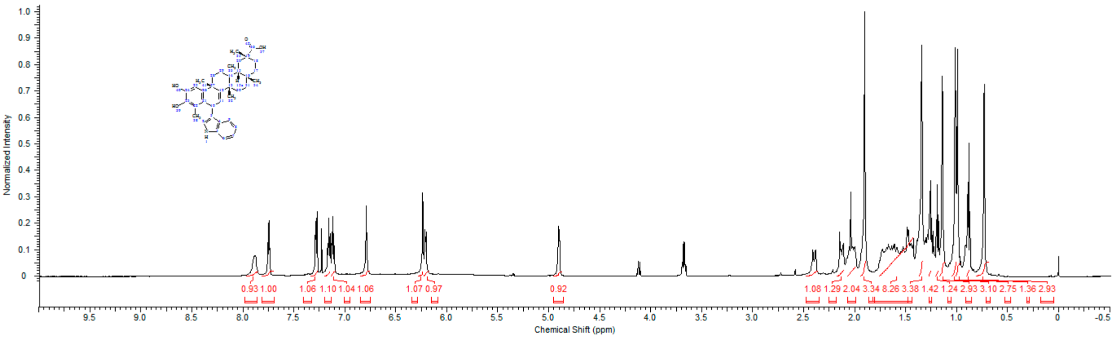

The ^{1}H NMR spectrum of compound 3a

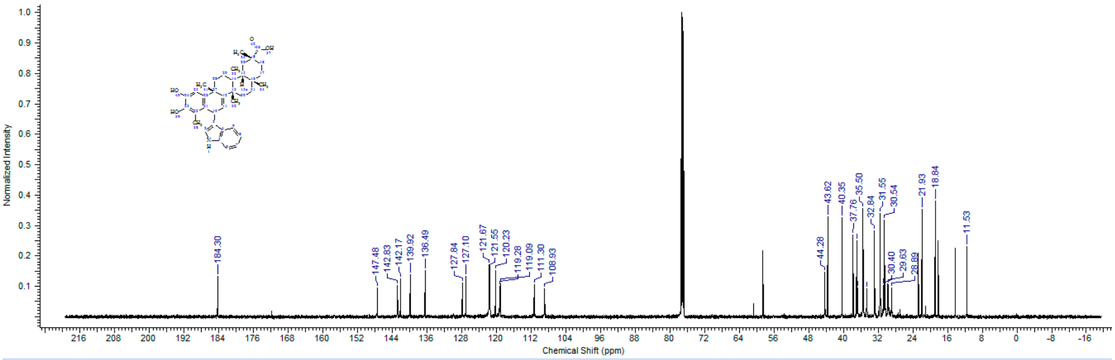

The ^{13}C NMR spectrum of compound 3a

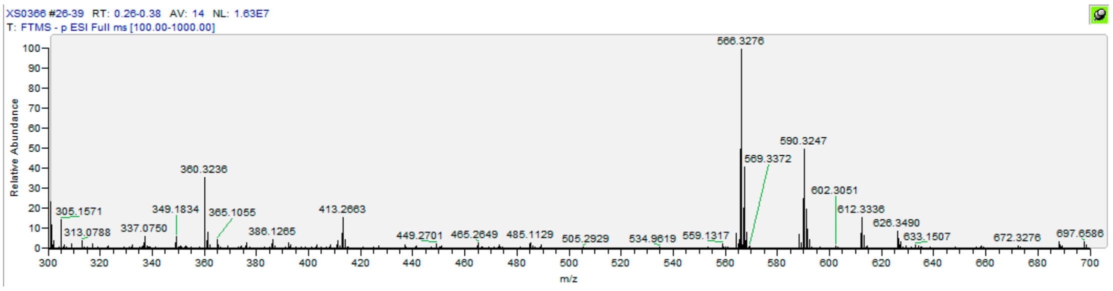

HRMS spectrum of compound 3a

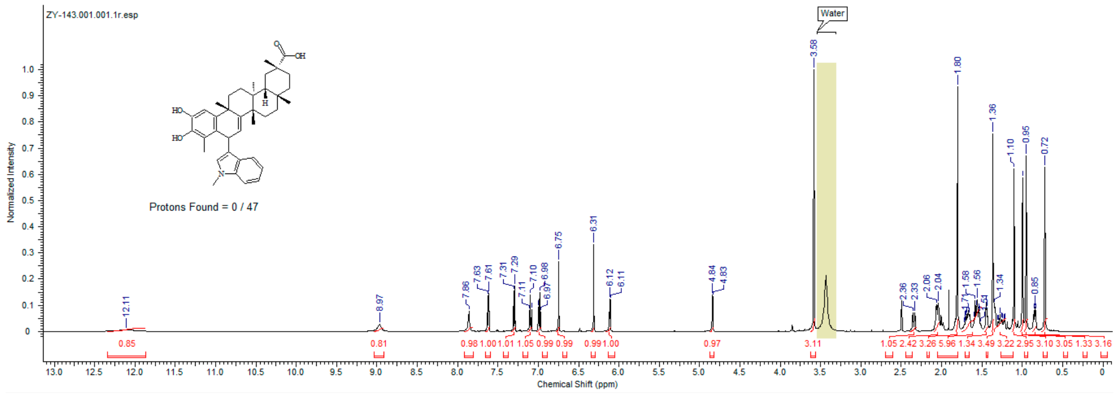

The ^{1}H NMR spectrum of compound 3b

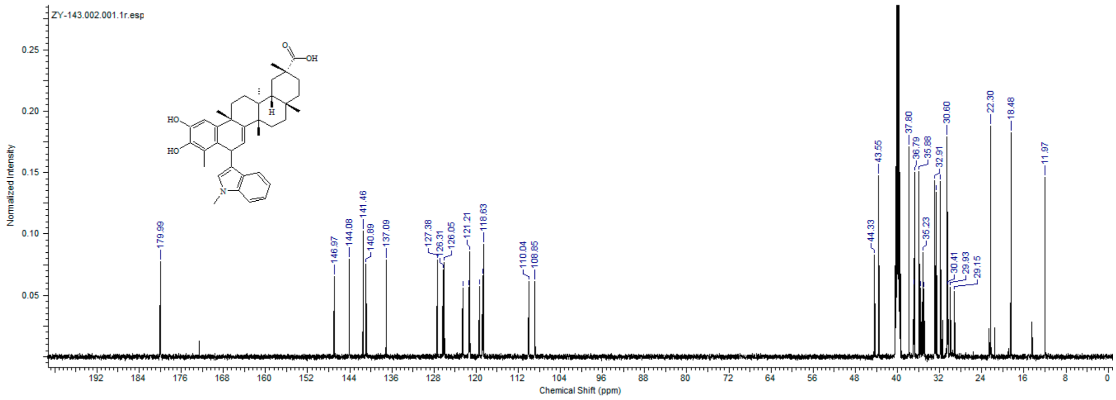

The ^{13}C NMR spectrum of compound 3b

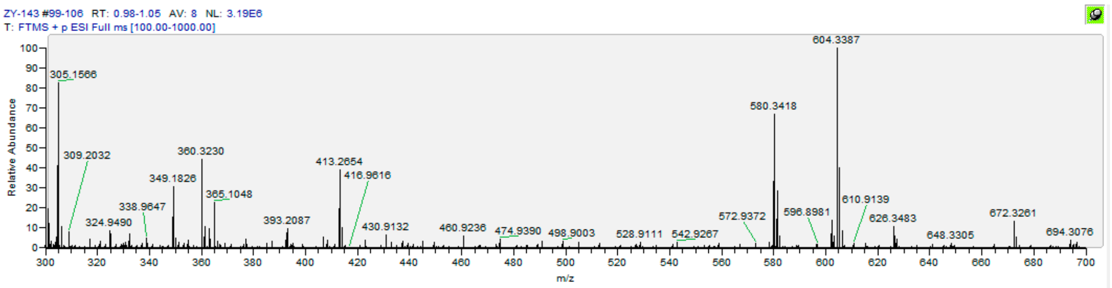

HRMS spectrum of compound 3b

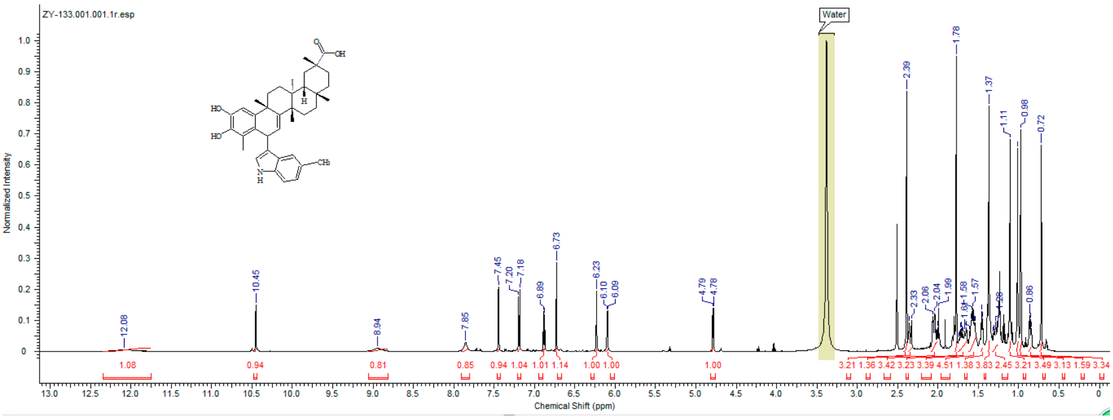

The ^{1}H NMR spectrum of compound 3c

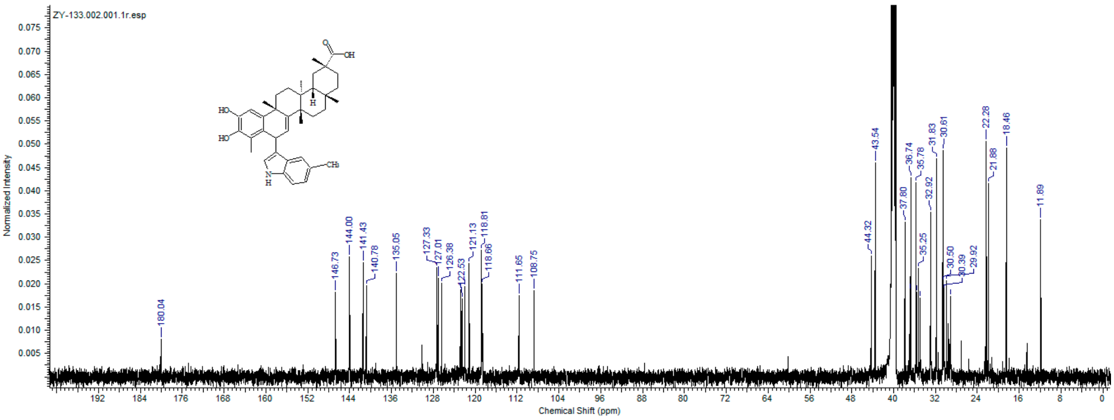

The ^{13}C NMR spectrum of compound 3c

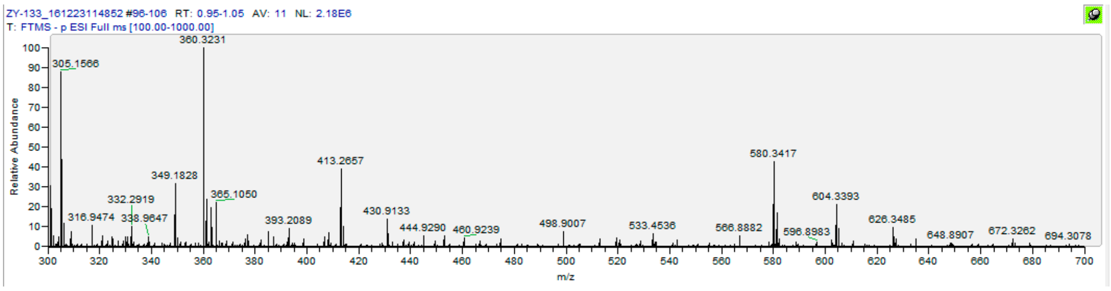

HRMS spectrum of compound 3c

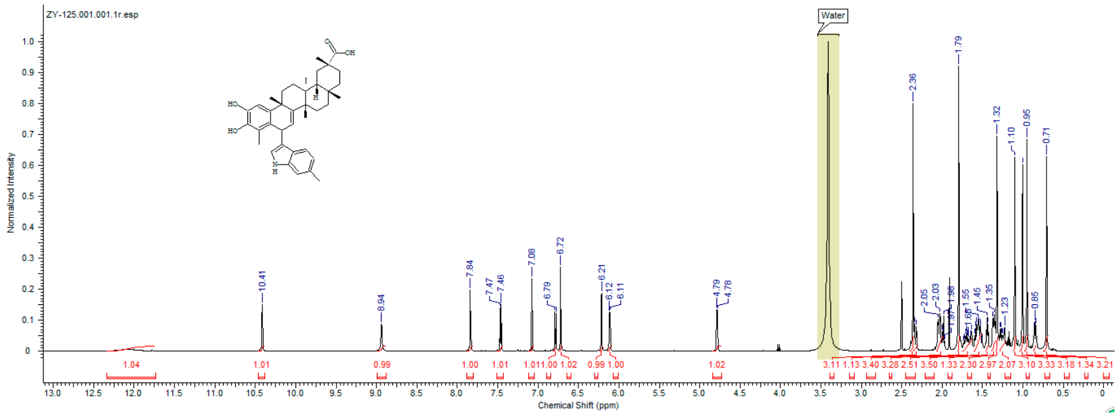

The ^{1}H NMR spectrum of compound 3d

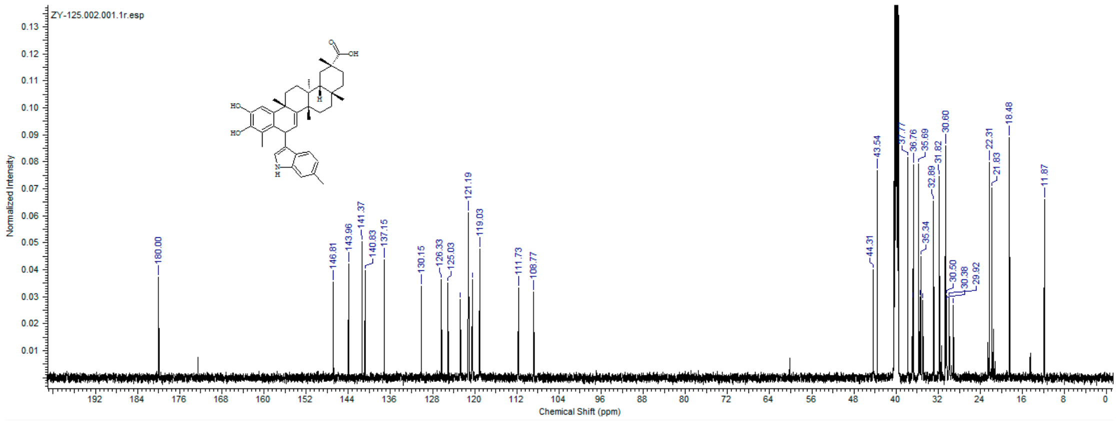

The ^{13}C NMR spectrum of compound 3d

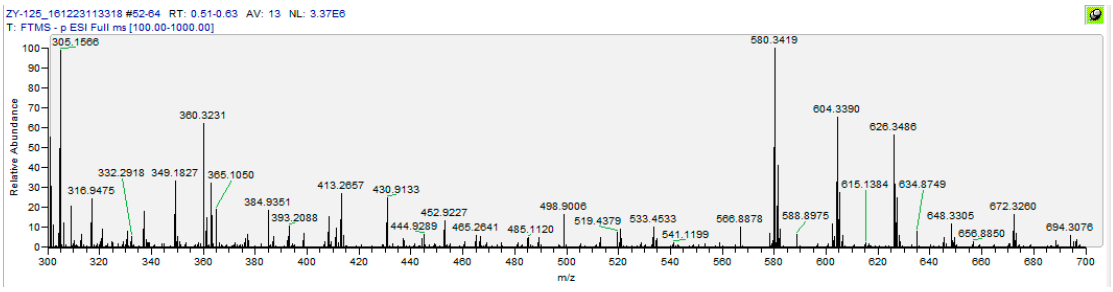

HRMS spectrum of compound 3d

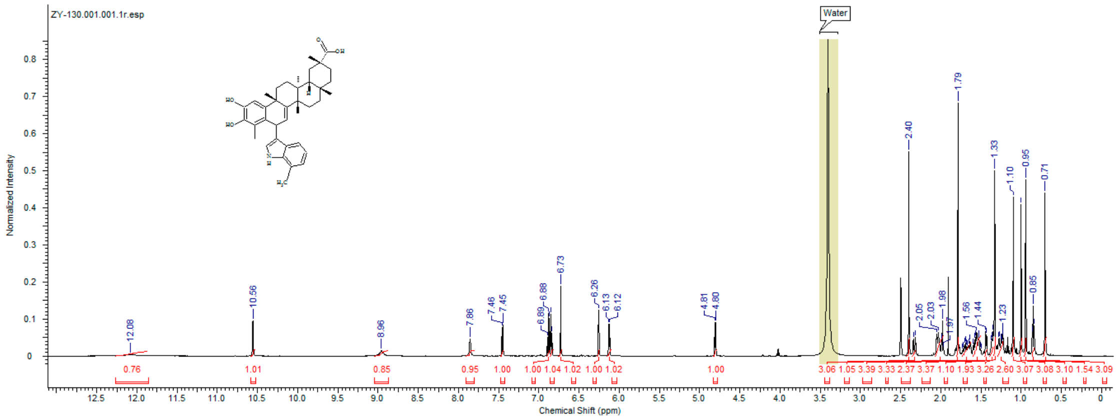

The ^{1}H NMR spectrum of compound 3e

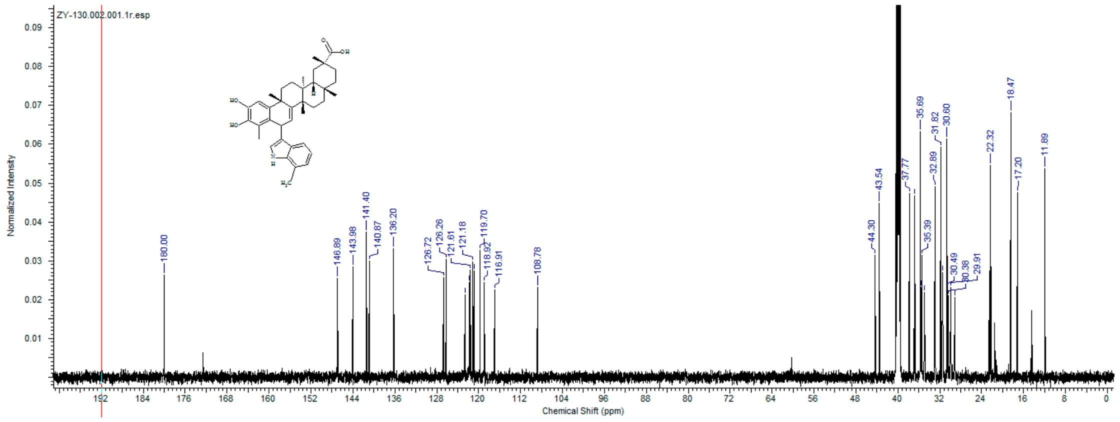

The ^{13}C NMR spectrum of compound 3e

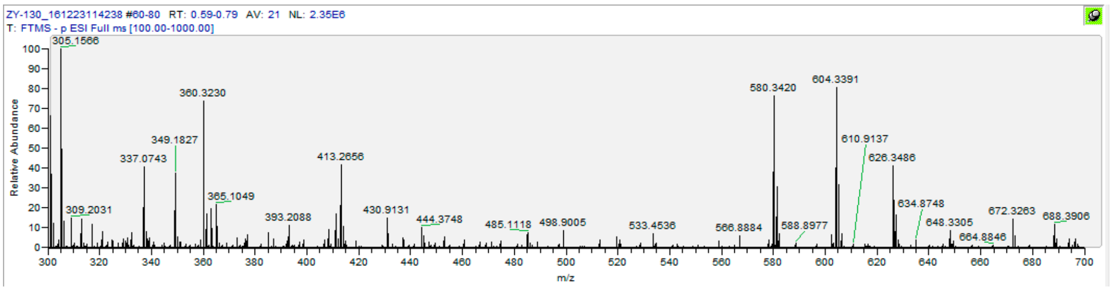

HRMS spectrum of compound 3e

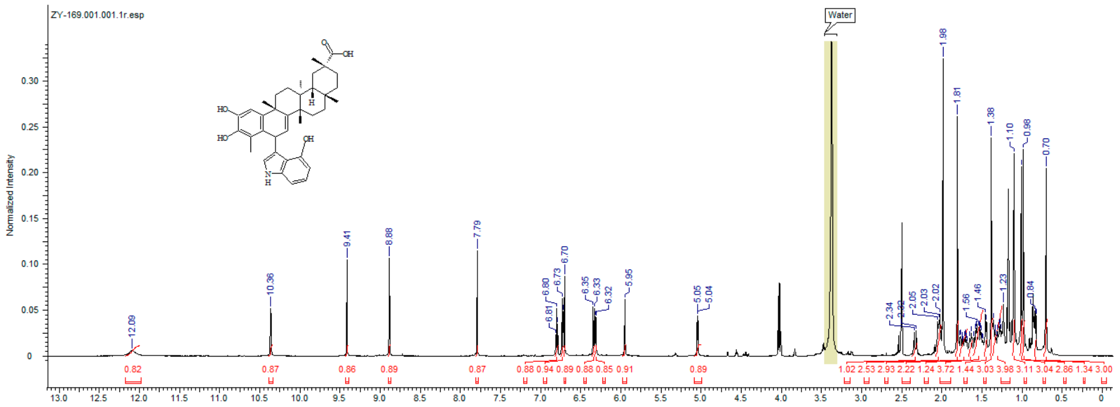

The ^{1}H NMR spectrum of compound 3f

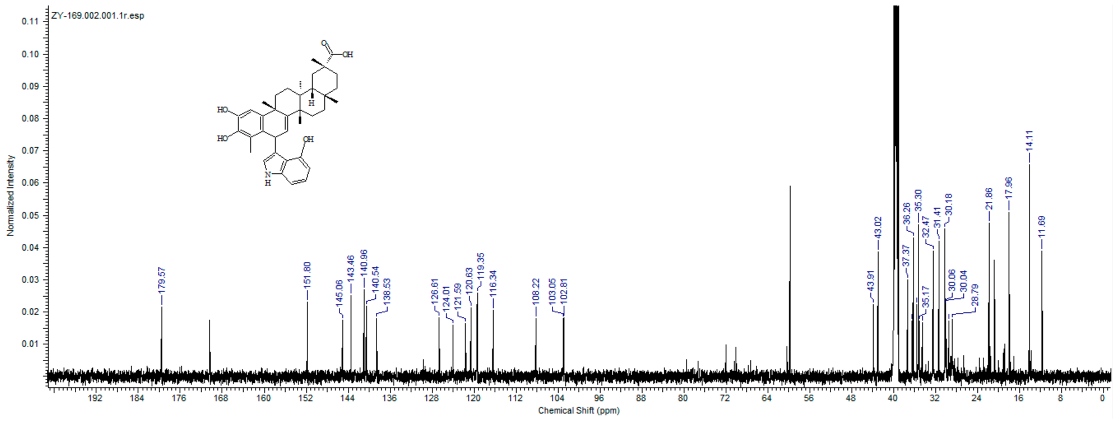

The ^{13}C NMR spectrum of compound 3f

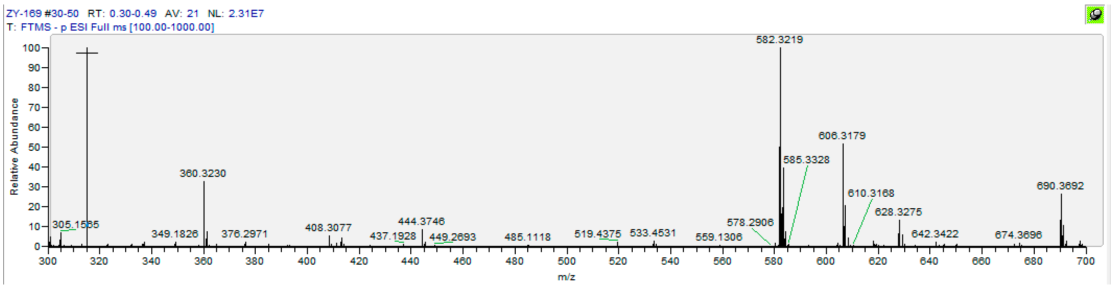

HRMS spectrum of compound 3f

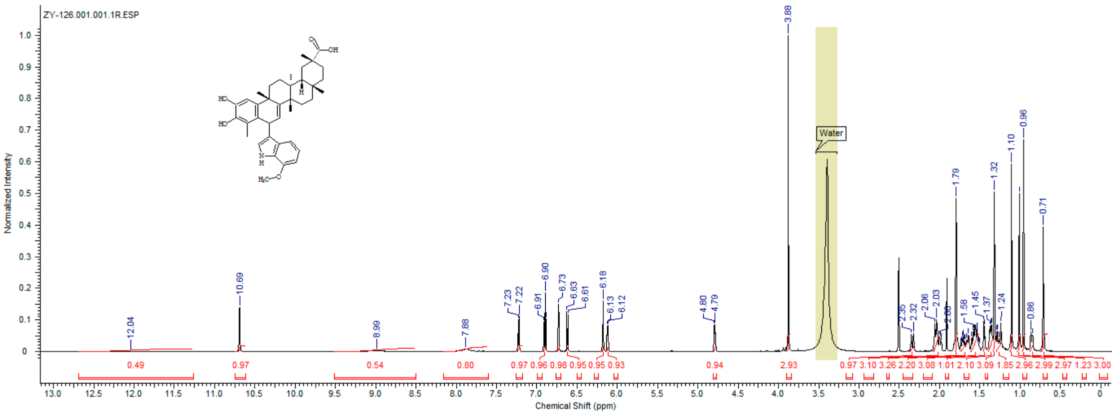

The ^{1}H NMR spectrum of compound 3h

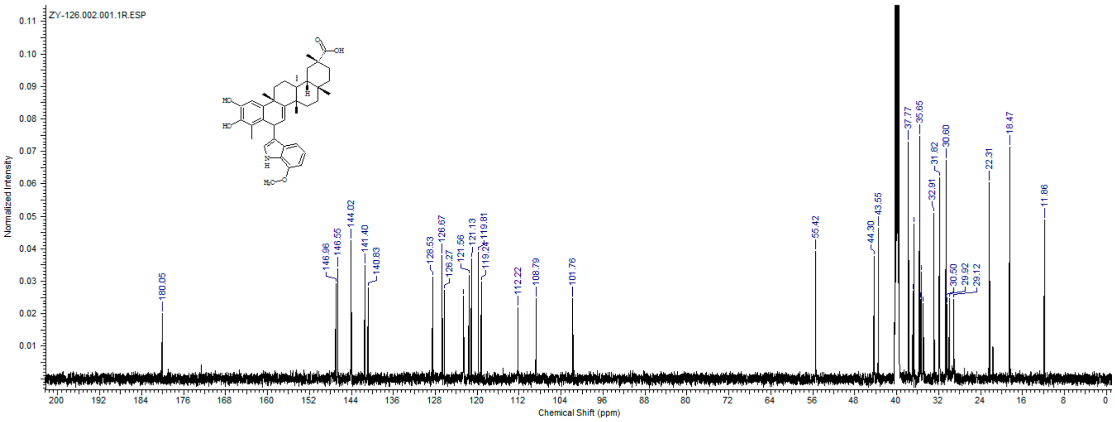

The ^{13}C NMR spectrum of compound 3h

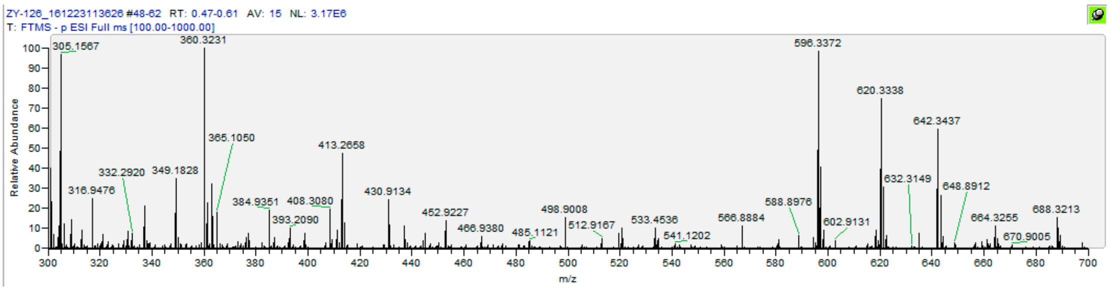

HRMS spectrum of compound 3h

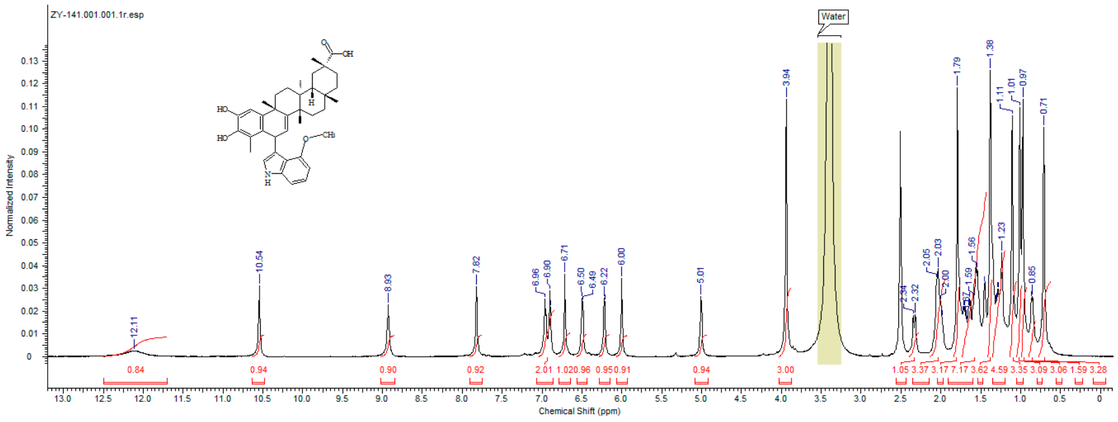

The ^{1}H NMR spectrum of compound 3i

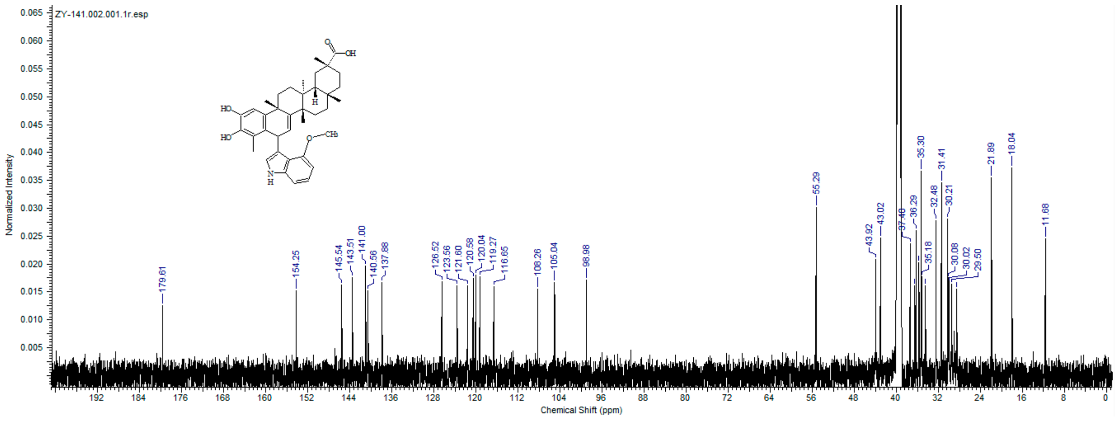

The ^{13}C NMR spectrum of compound 3i

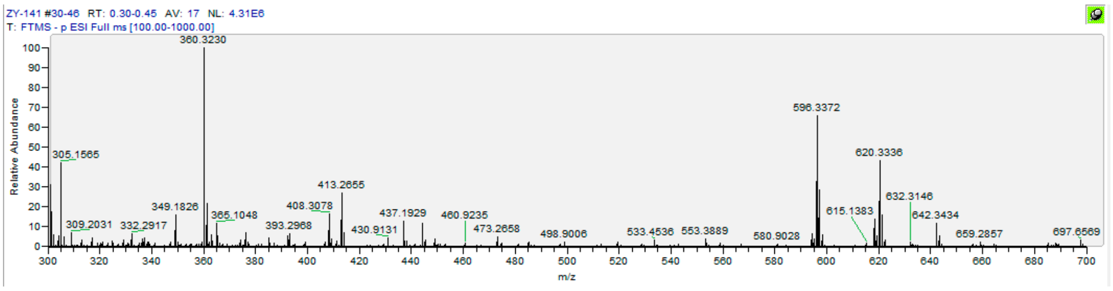

HRMS spectrum of compound 3i

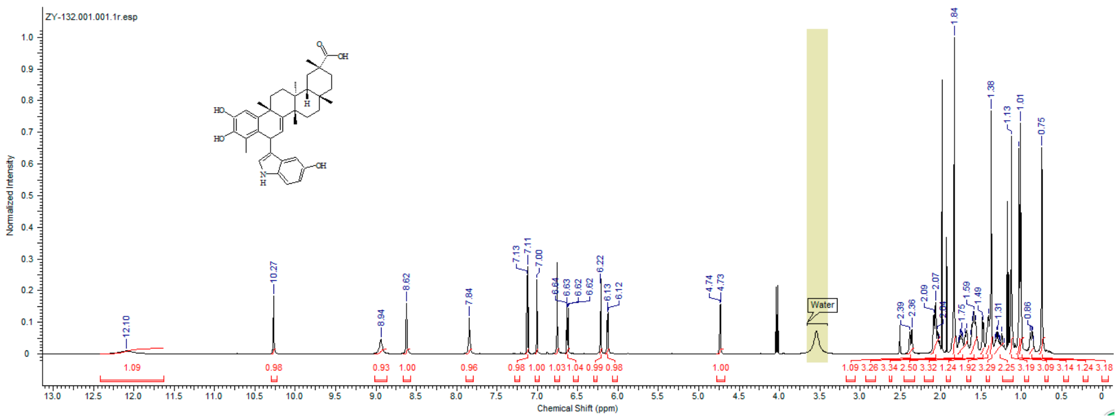

The ^{1}H NMR spectrum of compound 3j

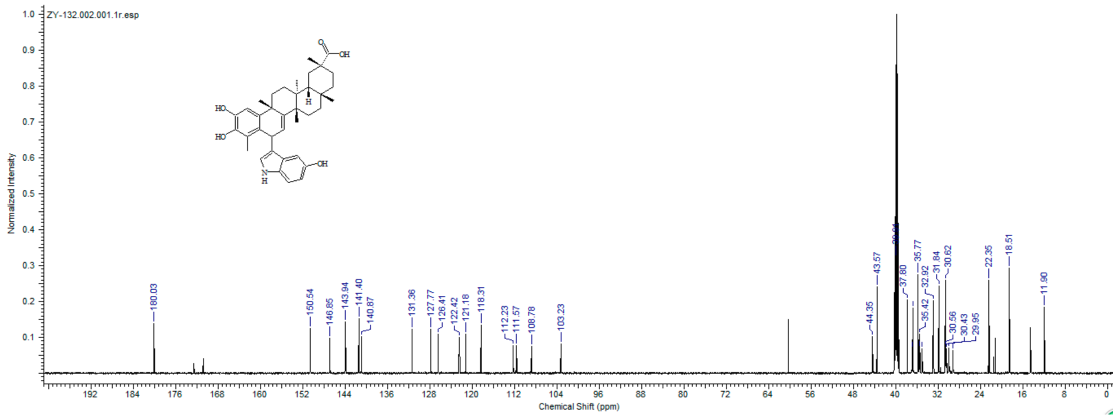

The ^{13}C NMR spectrum of compound 3j

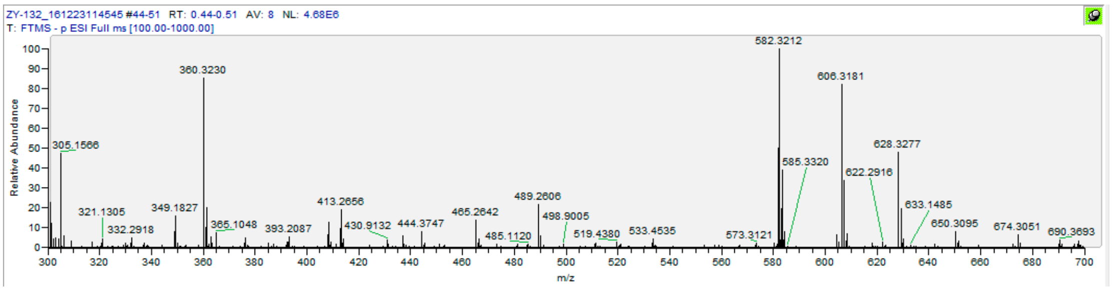

HRMS spectrum of compound 3j

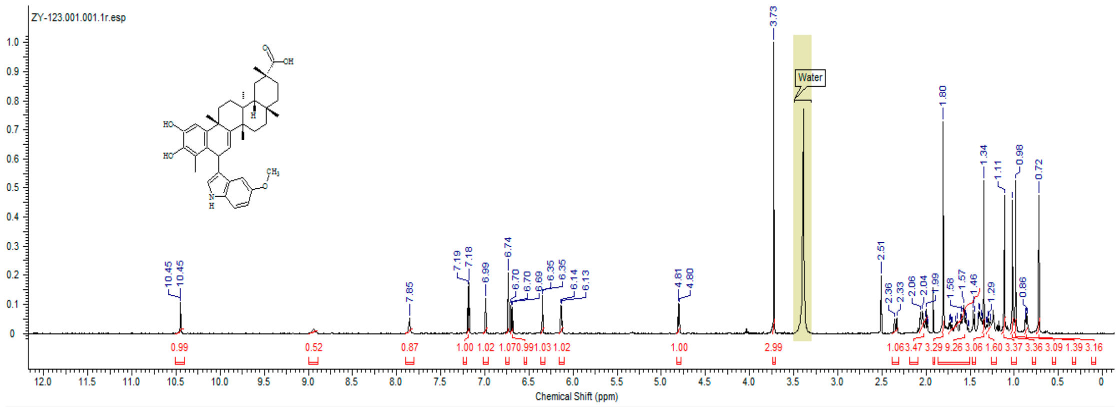

The ^{1}H NMR spectrum of compound 3k

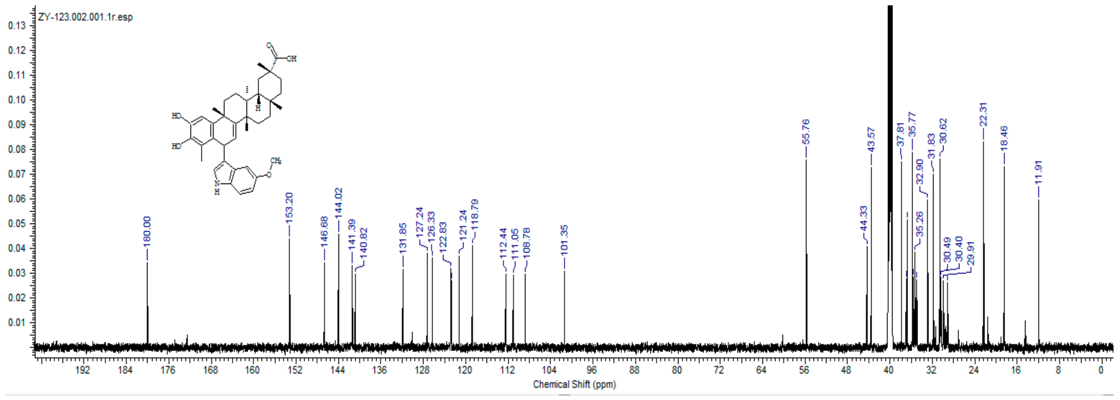

The ^{13}C NMR spectrum of compound 3k

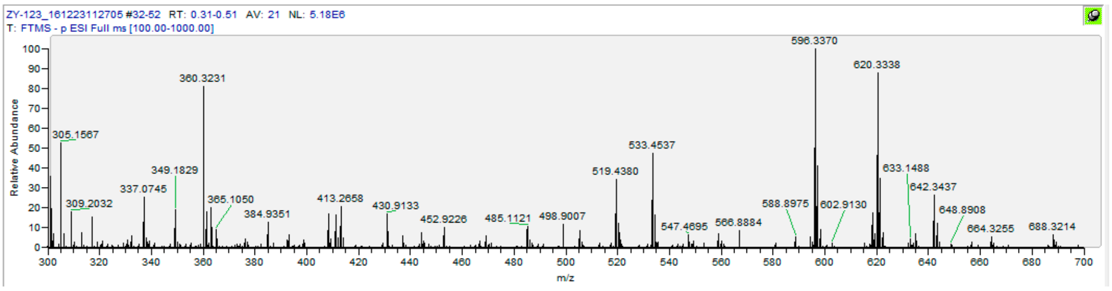

HRMS spectrum of compound 3k

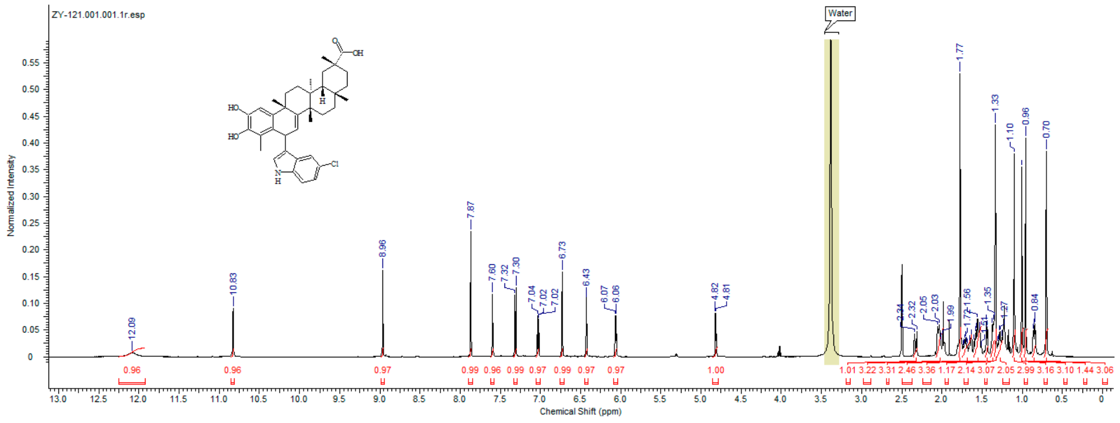

The ^{1}H NMR spectrum of compound 3l

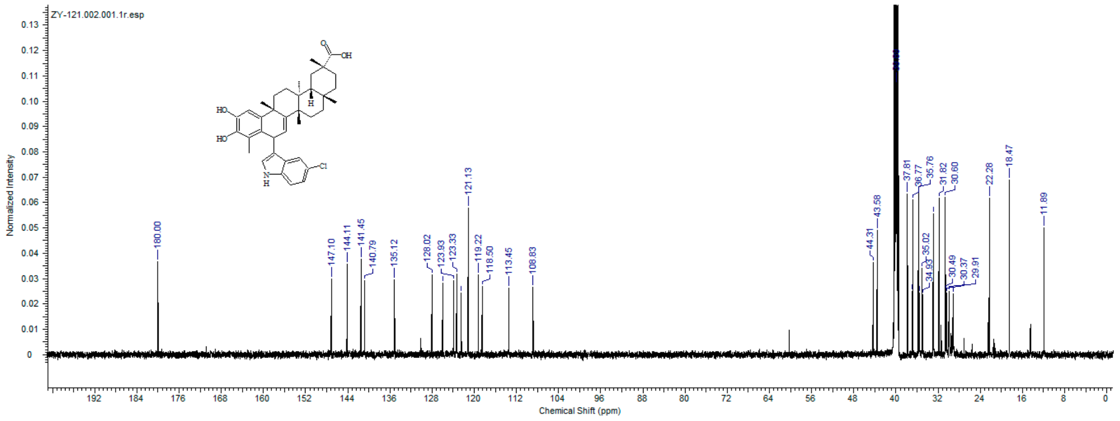

The ^{13}C NMR spectrum of compound 3l

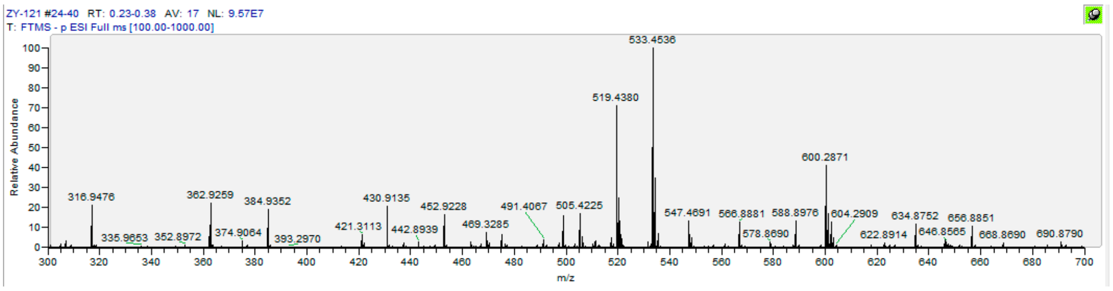

HRMS spectrum of compound 3l

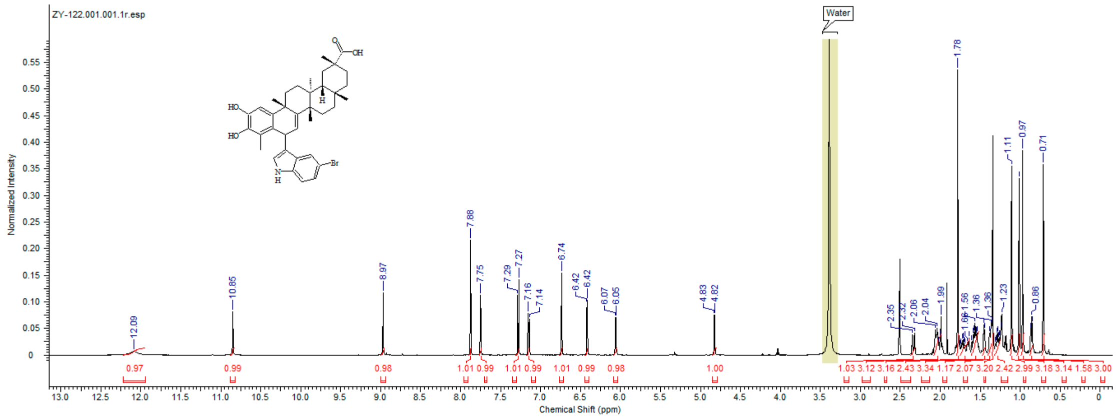

The ¹H NMR spectrum of compound 3m

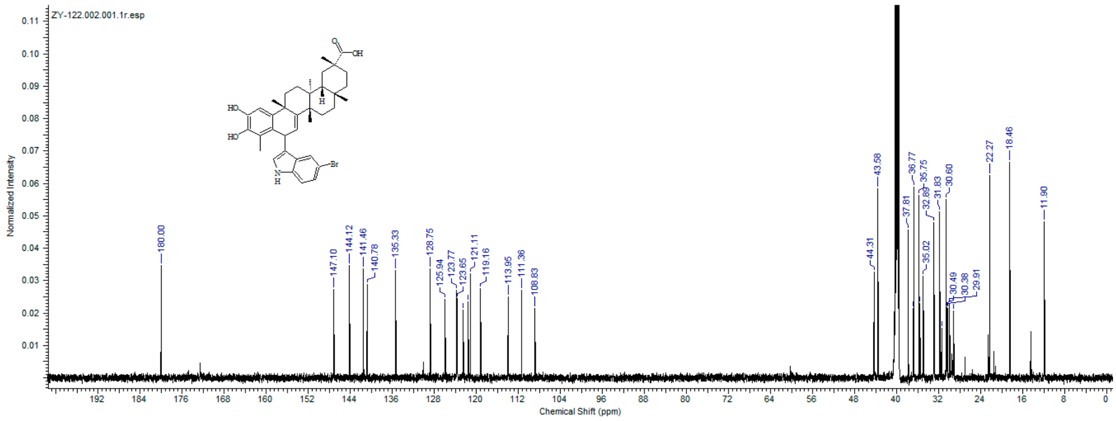

The ¹³C NMR spectrum of compound 3m

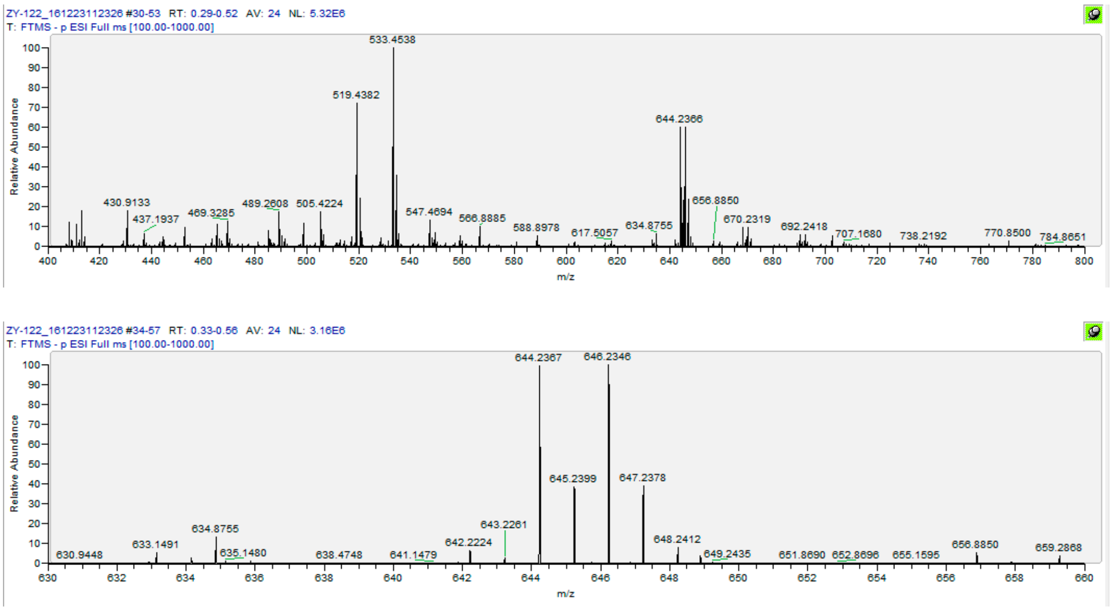

HRMS spectrum of compound 3m

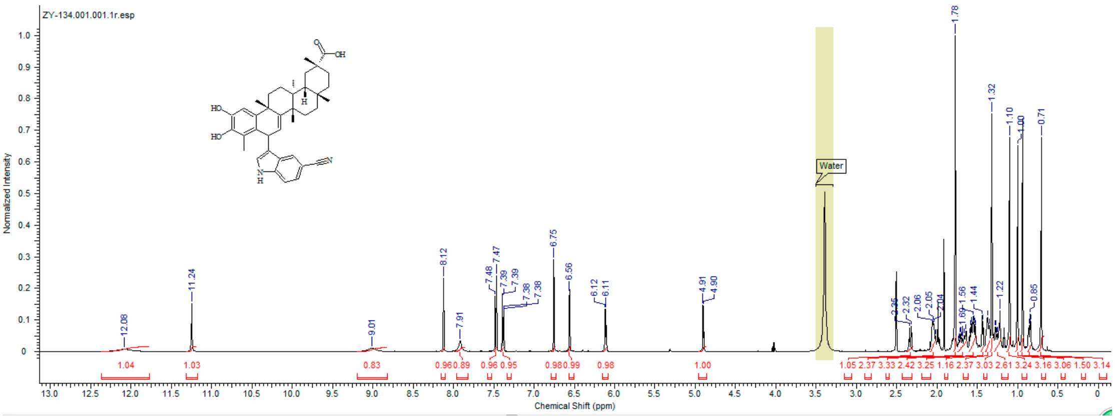

The ^{1}H NMR spectrum of compound 3n

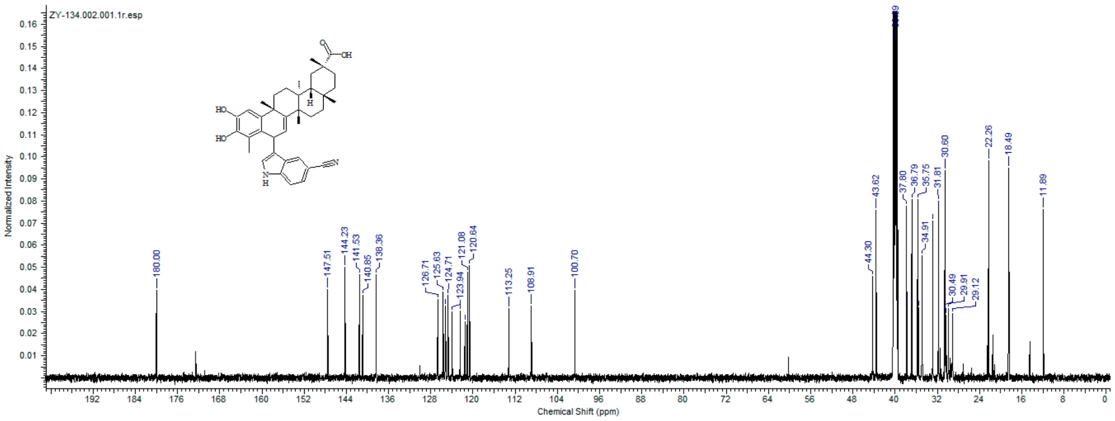

The ^{13}C NMR spectrum of compound 3n

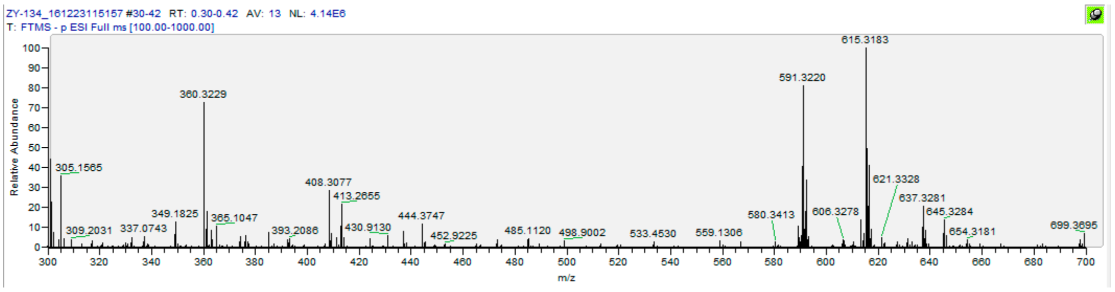

HRMS spectrum of compound 3n

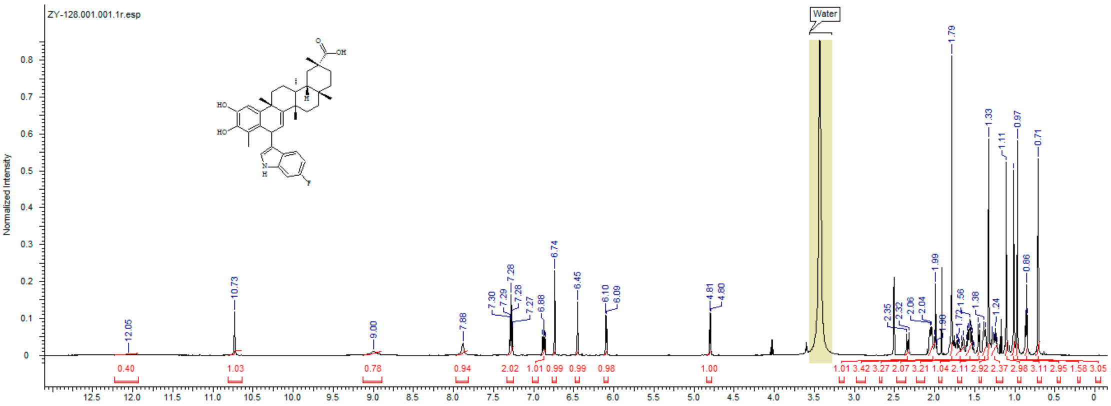

The ^{1}H NMR spectrum of compound 3o

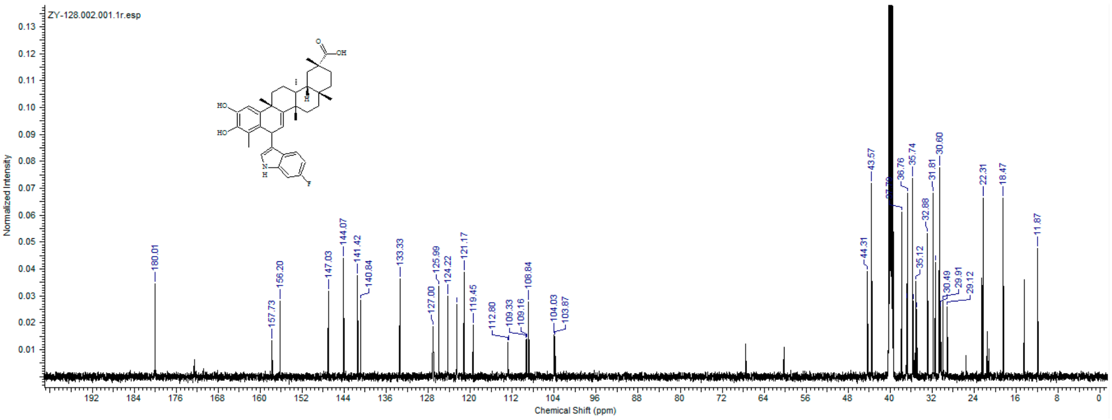

The ^{13}C NMR spectrum of compound 3o

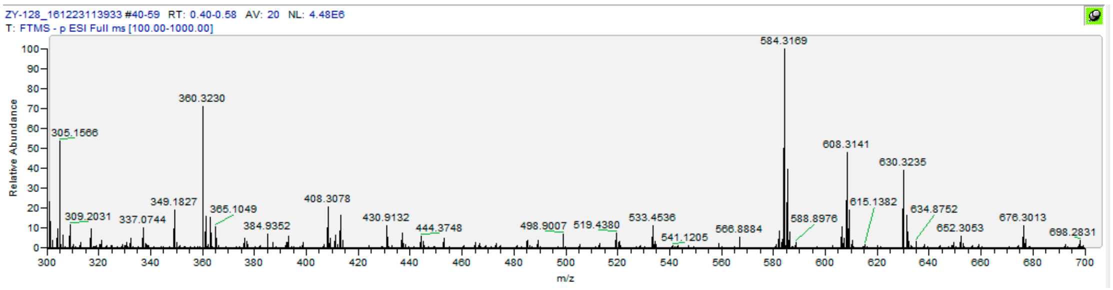

HRMS spectrum of compound 3o

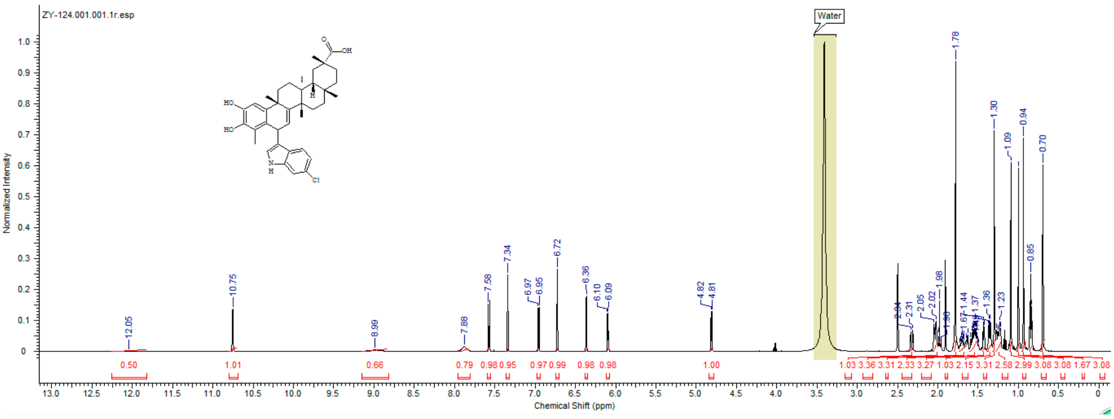

The ^{1}H NMR spectrum of compound 3p

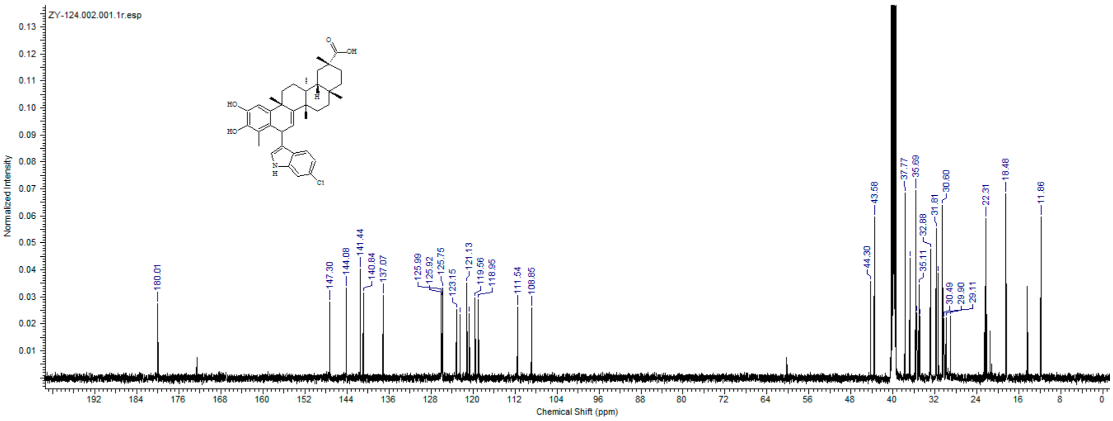

The ^{13}C NMR spectrum of compound 3p

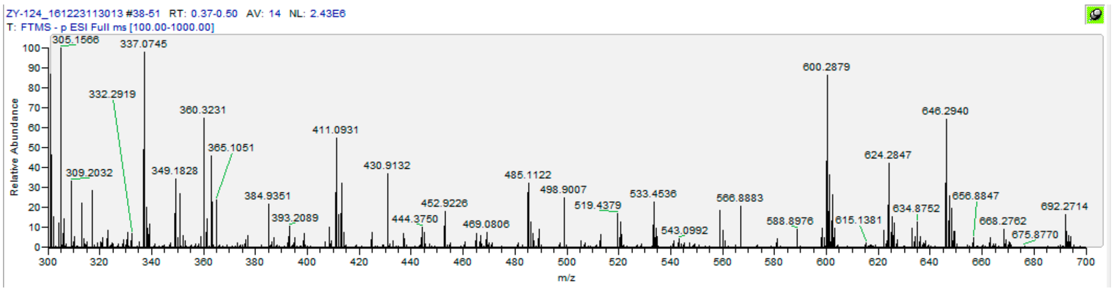

HRMS spectrum of compound 3p

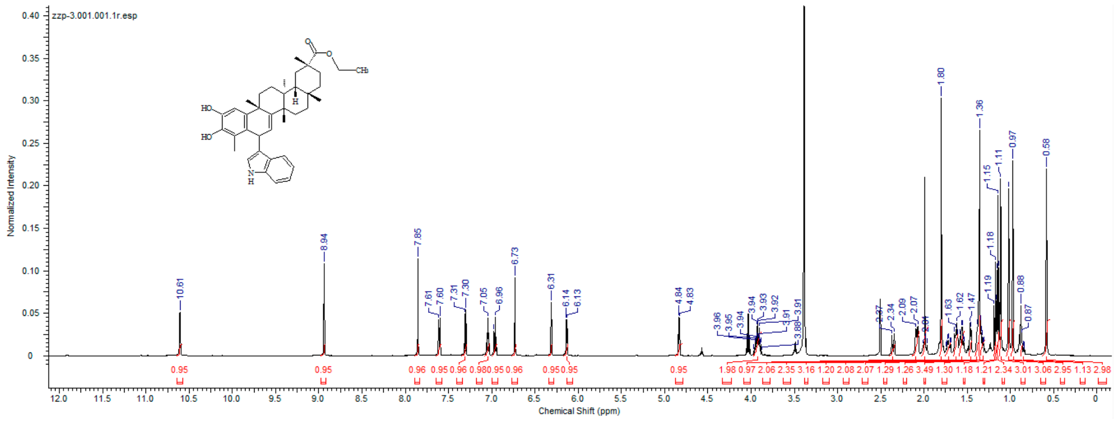

The ^{1}H NMR spectrum of compound 1a2b

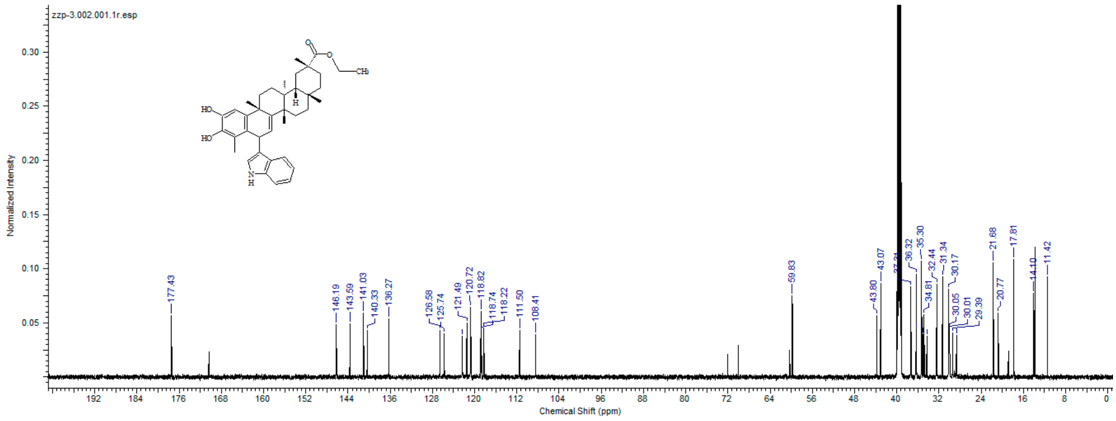

The ^{13}C NMR spectrum of compound 1a2b

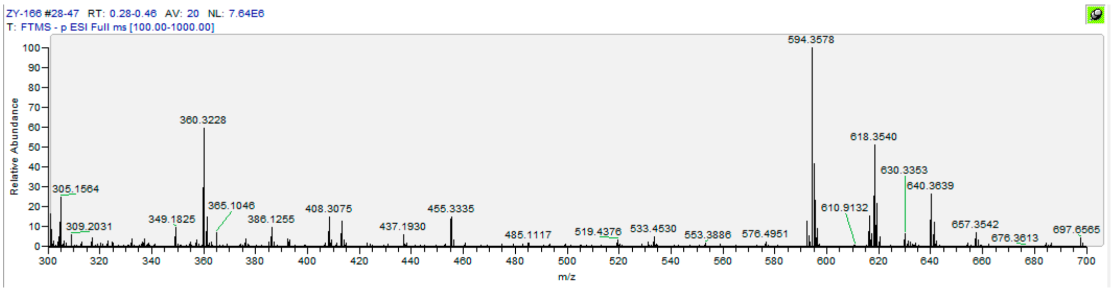

HRMS spectrum of compound 1a2b

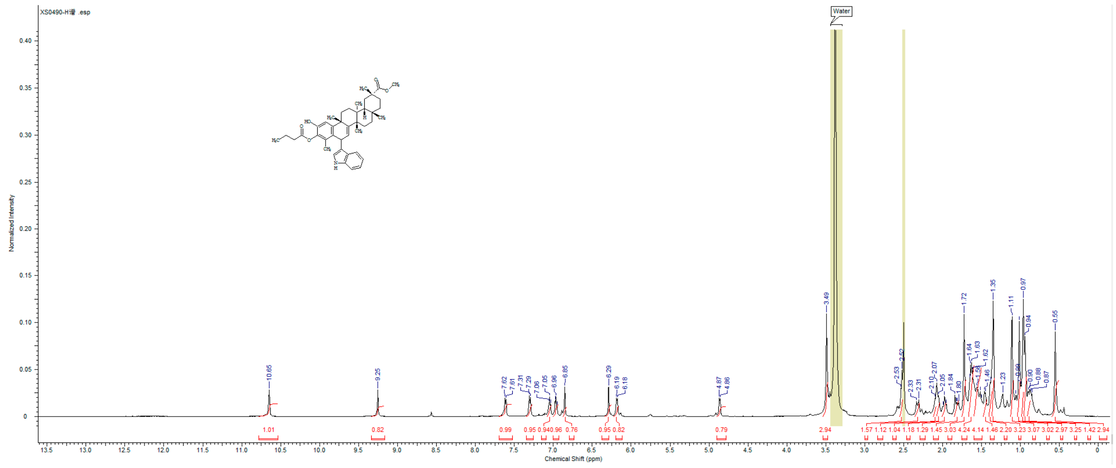

The ^{1}H NMR spectrum of compound 1a2c

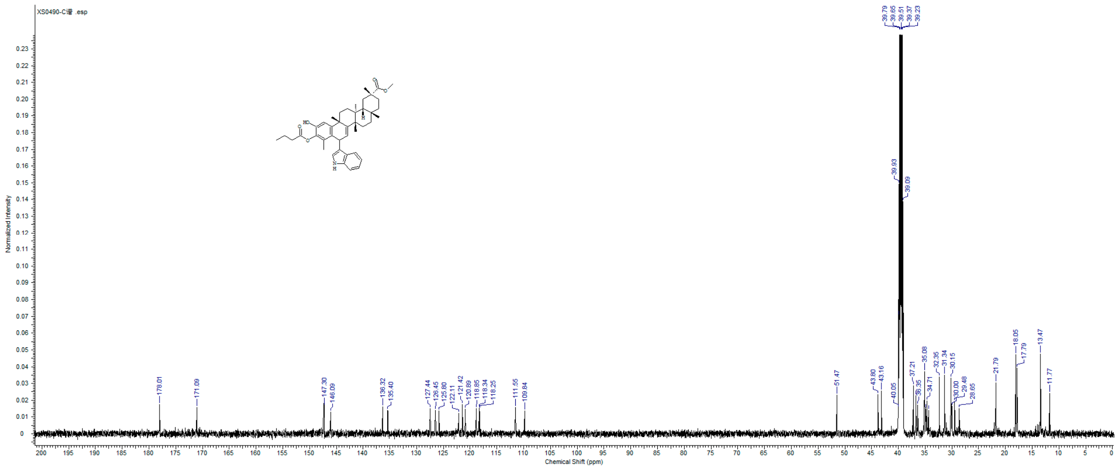

The ^{13}C NMR spectrum of compound 1a2c
